# Arrhythmias in Dengue: A Systematic Review and Meta-Analysis

**DOI:** 10.3390/pathogens15050497

**Published:** 2026-05-05

**Authors:** Darío S. López-Delgado, Mathias S. Renteros-Ramirez, Joshua Emmanuel Arteaga-Bolaños, Harold E. Vásquez-Ucros, Kevin Alexander Burbano-Castro, Valentina Reina-Melo, Jessica Niebles-Blanco, Nancy Calzada-Gonzales, Lysien I. Zambrano, Valmore Bermudez, Alfonso J. Rodriguez-Morales

**Affiliations:** 1 Hospital Universitario Departamental de Nariño, Pasto 520002, Colombia; 2 Facultad de Medicina, Universidad Cooperativa de Colombia, Pasto 520002, Colombia; joshua.arteagab@campusucc.edu.co (J.E.A.-B.);; 3 Facultad de Medicina, Universidad San Martin, Pasto 520002, Colombia; 4 Facultad de Medicina, Universidad Peruana de Ciencias Aplicadas, Lima 15023, Peru; mathias.renteros@gmail.com; 5 Facultad de Medicina, Universidad Nacional Hermilio Valdizan, Huánuco 10004, Peru; 6 Department of Morphological Sciences, School of Medical Sciences, Universidad Nacional Autónoma de Honduras, Tegucigalpa 11101, Honduras; 7 Centro de Investigaciones en Ciencias de la Vida, Facultad de Ciencias de la Salud, Universidad Simón Bolívar, Barranquilla 080002, Colombia; 8 Faculty of Health Sciences, Universidad Cientifica del Sur, Lima 15074, Peru; 9 Grupo de Investigación Biomedicina, Faculty of Medicine, Fundación Universitaria Autónoma de las Américas-Institución Universitaria Visión de las Américas, Risaralda 660003, Colombia

**Keywords:** dengue, cardiac arrhythmias, bradycardia, tachycardia, atrioventricular block, electrocardiography, systematic review and meta-analysis

## Abstract

Background: Cardiac involvement in dengue has been increasingly recognized, yet the true burden and spectrum of arrhythmias remain uncertain due to heterogeneous and fragmented evidence. We conducted a systematic review and meta-analysis to estimate the proportion of cardiac arrhythmias in patients with dengue and to describe the distribution of major arrhythmia subtypes. Methods: We searched PubMed/MEDLINE, EMBASE, LILACS, Global Index Medicus, and Google Scholar from inception to November 2025 without language restrictions. Observational studies reporting the number of dengue patients evaluated for arrhythmias and the number with at least one rhythm disturbance were included. Random-effects generalized linear mixed models with a logit transformation were used to estimate pooled proportion with 95% confidence intervals (CIs). Subgroup analyses were performed by age group. Risk of bias was assessed using the Joanna Briggs Institute tool, and certainty of evidence was evaluated with GRADE. Results: Thirty-five studies, including 6948 patients, were analyzed. The pooled proportion of any arrhythmia was 24.48% (95% CI 17.54–33.07), with a higher proportion in adults (30.00%) than in children (10.73%). Sinus bradycardia (11.84%) and sinus tachycardia (10.63%) were the most frequent abnormalities. Atrioventricular block was uncommon (1.33%). Between-study heterogeneity was high for most outcomes. No significant small-study effects were detected. Conclusions: Cardiac arrhythmias occur in approximately one in four patients with dengue, predominantly as sinus rate abnormalities. While often transient, these findings support the role of baseline and risk-based ECG monitoring, particularly in hospitalized adults and patients with severe disease.

## 1. Introduction

Dengue is a major arboviral infection worldwide. It is caused by the dengue virus (DENV), a positive-sense single-stranded RNA virus of the Flaviviridae family with four main serotypes (DENV-1 to DENV-4), and is transmitted predominantly by *Aedes aegypti* and *Aedes albopictus* mosquitoes [1]. It is endemic in more than 100 countries, particularly in tropical and subtropical regions, with an estimated 400 million infections annually, and a substantial global disease burden [2,3,4,5,6].

Beyond its classical clinical manifestations, cardiovascular involvement in dengue—including myocarditis, conduction disturbances, and arrhythmias—has gained increasing recognition. Although traditionally considered uncommon, accumulating observational evidence suggests that cardiac complications may be more frequent, particularly among hospitalized patients and those with severe disease [7,8]. Reported abnormalities range from sinus bradycardia and tachycardia to atrioventricular block and other conduction disturbances, with potential hemodynamic and prognostic implications.

Despite this growing recognition, the evidence on arrhythmias in dengue remains fragmented. Most studies are single-center observational cohorts with heterogeneous designs, variable electrocardiogram (ECG) monitoring strategies, and inconsistent definitions of rhythm disturbances. In addition, prior reviews have been largely narrative [8]. Consequently, the overall burden, subtype distribution, age-related differences, and clinical relevance of arrhythmias in dengue are not well established.

To address this gap, we conducted a systematic review and meta-analysis to estimate the pooled proportion of cardiac arrhythmias in patients with DENV infection, characterize the main arrhythmia subtypes, and explore differences between adults and children.

## 2. Methods

### 2.1. Study Design and Reporting

We conducted a systematic review and meta-analysis to estimate the proportion and spectrum of cardiac arrhythmias among patients with dengue infection. The review was designed a priori according to the Population, Exposure, Outcome, and Time (PEOT) framework, and the protocol was registered with the International Prospective Register of Systematic Reviews (PROSPERO: 2025 CRD420251207231). Reporting follows the Preferred Reporting Items for Systematic Reviews and Meta-Analyses PRISMA 2020 statement [9].

### 2.2. Eligibility Criteria

We included observational studies (cross-sectional, cohort, and case–control studies, as well as case series with ≥10 participants) that met all of the following criteria:

1. Population: Patients of any age with confirmed or probable dengue infection, diagnosed either by laboratory methods (RT-PCR, NS1 antigen, or serology with IgM/IgG seroconversion) or by clinical criteria consistent with World Health Organization (WHO 1997 or 2009) definitions.
2. Outcome: Reporting the number of patients with at least one cardiac arrhythmia during the index dengue episode, detected by 12-lead electrocardiography (ECG), continuous cardiac monitoring, or Holter recording. Cardiac arrhythmia was defined as any rhythm disturbance documented by 12-lead ECG, serial ECG, telemetry, or Holter monitoring during the index dengue episode. For adults, sinus tachycardia was defined as a resting sinus rhythm >100 beats/min, and sinus bradycardia as a resting sinus rhythm <60 beats/min unless the original study used a different prespecified threshold. For pediatric populations, tachycardia and bradycardia were accepted according to age-adjusted thresholds reported by the study authors; when not explicitly defined, they were interpreted against established pediatric reference ranges, recognizing that normal heart rate varies by age. AV block and non-sinus supraventricular or ventricular arrhythmias were accepted only when explicitly electrocardiographically documented. Because diagnostic definitions varied across studies, we did not retrospectively reclassify arrhythmias; instead, we extracted author-defined outcomes, recorded the monitoring modality and diagnostic criteria when available, and addressed this heterogeneity through risk-of-bias assessment, subgroup analyses, and cautious interpretation.
3. Design: Studies with a clearly defined denominator (total number of dengue patients assessed for rhythm disturbances) allowing calculation of a proportion. We included cross-sectional, cohort, and other observational studies, as well as case-series-type reports, only when they enrolled a clearly defined series of patients with DENV infection and provided a valid denominator for the number of patients assessed for cardiac arrhythmias. Studies were classified according to their original design and sampling strategy, rather than the number of arrhythmia events reported. We excluded reports restricted to patients already selected for cardiac complications or studies without a clear denominator.
4. Setting: Any clinical setting (outpatient, emergency department, ward, or intensive care unit) and any country or income level.

We excluded case reports and small case series (<10 patients), review articles, editorials, conference abstracts without extractable data, animal or in vitro studies, and studies that did not report either the total number of patients evaluated or the number with arrhythmias. When multiple publications reported overlapping populations, we included the report with the most complete or recent data.

No restriction by time nor language was made.

### 2.3. Information Sources and Search Strategy

A comprehensive search of the biomedical literature was conducted in PubMed/MEDLINE, EMBASE, LILACS, Global Index Medicus, and Google Scholar from database inception to the date of the last search in November 2025. The search strategy combined controlled vocabulary and free-text terms related to dengue (e.g., “dengue”, “dengue hemorrhagic fever”, “dengue shock syndrome”) and cardiac rhythm disturbances (e.g., “arrhythmia”, “bradycardia”, “tachycardia”, “atrioventricular block”, “conduction disorder”, “electrocardiography”). The strategy was adapted for each database, and no restrictions on language, publication status, or publication date were applied.

To identify additional eligible studies, we manually screened the reference lists of all included articles and relevant reviews and tracked citations of key studies in Google Scholar. The complete search strategy is shown in the Supplementary Materials.

### 2.4. Study Selection

Three reviewers (KABC, VRM, and JNB) independently screened titles and abstracts and assessed full texts for eligibility. Data extraction was performed independently by MSRR, JEAB, NCG and HEVU using a standardized, piloted extraction form. Disagreements during study selection or data extraction were resolved by consensus with DSLD, VB and AJRM. Two reviewers independently screened titles and abstracts to exclude clearly irrelevant articles. The full texts of potentially eligible reports were then assessed in duplicate against the inclusion and exclusion criteria. Discrepancies were resolved through discussion and, when necessary, consultation with a third reviewer. Reasons for exclusion at the full-text stage were documented. The study selection process is summarized in a PRISMA flow diagram.

### 2.5. Data Extraction

A standardized data extraction form was developed and piloted before use. Two reviewers independently extracted data from each included study. Extracted variables comprised:

- Study characteristics: first author, year of publication, country, study design, and clinical setting.
- Population characteristics: sample size, age group (children, adults, or mixed), definitions and diagnostic criteria for dengue, and severity categories when reported. Age group was extracted for each study. For subgroup analyses, studies were classified as pediatric if they enrolled participants aged <18 years and as adult if they enrolled participants aged ≥18 years. When the original study used a different age boundary, that definition was retained and is specified where relevant.
- Outcome assessment: method and timing of ECG or rhythm monitoring, definition of arrhythmia, and whether assessment was systematic or triggered by symptoms.
- Outcomes: total number of dengue patients evaluated for rhythm disturbances (denominator) and number of patients with at least one arrhythmia (numerator). When available, we separately extracted the number of patients with bradycardia, tachycardia, AV block, and other arrhythmias.

When data were missing or unclear, we attempted to infer numerators and denominators from text, tables, or figures. If this was not possible, the study was retained in the qualitative synthesis but excluded from the corresponding quantitative analysis for that specific outcome. Disagreements in data extraction were resolved by consensus.

### 2.6. Outcomes

The primary outcome was the pooled proportion of patients with any cardiac arrhythmia during the index illness with dengue.

Secondary outcomes included the pooled proportion of specific arrhythmia subtypes (bradycardia, tachycardia, AV block, and other arrhythmias) and subgroup analyses according to age group (adults vs. children).

### 2.7. Risk of Bias Assessment

Risk of bias for proportion outcomes was assessed independently by two reviewers using an adapted version of the Joanna Briggs Institute (JBI) critical appraisal checklist for studies reporting proportion data [10]. The tool evaluates sampling methods, sample size, the description of the population and setting, the validity of the condition measurement, the reliability of the outcome assessment, and the appropriateness of the statistical analysis. Each domain was rated as low, high, or unclear risk of bias. Overall risk of bias was summarized qualitatively across studies; no study was excluded solely based on risk of bias.

### 2.8. Certainty of Evidence

Certainty of the evidence for the primary and secondary proportion outcomes was assessed using the Grading of Recommendations Assessment, Development and Evaluation (GRADE) approach [11]. For each outcome, two reviewers independently rated the certainty of the evidence as high, moderate, low, or very low, considering risk of bias, inconsistency, indirectness, imprecision, and potential publication bias. Disagreements were resolved through discussion and, when necessary, consultation with a third reviewer.

### 2.9. Statistical Analysis

For each study, proportions were calculated as the number of patients with the outcome of interest divided by the total number of patients with DENV infection evaluated for arrhythmias. Meta-analyses were performed when at least three studies reported the same outcome with sufficient information for extraction.

The primary meta-analytic approach used a random-effects generalized linear mixed model (GLMM) with a logit transformation of proportions to obtain pooled proportion estimates and 95% confidence intervals (CIs). This approach was selected because it is well-suited for single-arm proportion meta-analyses, particularly when event proportions vary widely across studies or approach 0 or 1, and it avoids the need for arbitrary continuity corrections. The GLMM was used to pool the overall proportion of any arrhythmia, the major arrhythmia subtypes, and the predefined subgroup analyses.

Between-study heterogeneity was assessed using the Cochran Q statistic (with the corresponding *p*-value), the between-study variance (τ^2^), and the I^2^ statistic, which represents the proportion of total variability attributable to heterogeneity rather than chance. I^2^ values of approximately 25%, 50%, and 75% were interpreted as low, moderate, and high heterogeneity, respectively.

Prespecified subgroup analyses of the primary outcome were performed by age group, geographic region, and diagnostic confirmation status when sufficient data were available. Studies were classified as pediatric if they enrolled participants aged <18 years and as adult if they enrolled participants aged ≥18 years; when the original study used a different age cutoff, the authors’ definition was retained. Geographic subgrouping was based on the study setting reported in the original publication. Studies were also categorized according to whether DENV infection was clearly laboratory confirmed or not.

To evaluate the robustness of the primary findings, we performed several sensitivity analyses. First, we repeated the meta-analysis for the primary outcome using a random-effects inverse-variance model with Freeman–Tukey double-arcsine transformation. This analysis was performed to assess the impact of the pooling framework and transformation choice on the pooled estimate. Second, we assessed influential studies using a Baujat plot. Based on this analysis, additional sensitivity analyses were conducted by excluding the studies by Baqi et al. and La-Fontaine-Terry et al., individually and jointly, to evaluate their influence on the pooled proportion and heterogeneity.

Small-study effects and potential publication bias for the primary outcome were explored visually using funnel plots and formally assessed with Egger’s regression test of the intercept. A two-sided *p*-value < 0.05 was considered to indicate statistically significant funnel plot asymmetry.

All analyses were performed in R version 4.5.3 (R Foundation for Statistical Computing, Vienna, Austria) using the meta package version 8.2-1 to pool proportions and generate forest, funnel, and Baujat plots.

For each study, proportions were calculated as the number of patients with the outcome of interest divided by the total number of dengue patients evaluated for arrhythmias. Meta-analyses were conducted when at least three studies reported the same outcome.

The primary meta-analytic approach used a random-effects generalized linear mixed model (GLMM) with a logit transformation of proportions to obtain pooled proportion estimates and 95% confidence intervals (CIs). This model naturally accommodates studies with very low or very high proportions and avoids the need for arbitrary continuity corrections. The same method was applied to synthesize the proportion of any arrhythmia, each arrhythmia subtype, and the age-based subgroups.

Between-study heterogeneity was quantified using the Cochran Q statistic (with the corresponding *p*-value), the between-study variance (τ^2^), and the I^2^ statistic, which represents the proportion of total variability due to heterogeneity rather than sampling error. We interpreted I^2^ values of approximately 25%, 50%, and 75% as low, moderate, and high heterogeneity, respectively.

As a sensitivity analysis, we repeated the meta-analysis for the primary outcome (overall proportion of any arrhythmia) using the Freeman–Tukey double-arcsine transformation with a random-effects inverse-variance model. This analysis was planned to evaluate the robustness of the results to the choice of transformation for proportion data.

Small-study effects and potential publication bias for the primary outcome were explored visually by funnel plots and formally using Egger’s regression test of the intercept. A *p*-value < 0.05 was considered to indicate statistically significant funnel plot asymmetry. All analyses were performed using R (R Foundation for Statistical Computing, Vienna, Austria) with the “meta” package to pool proportions and generate forest and funnel plots.

## 3. Results

### 3.1. Search Results and Study Selection

**Figure 1:**
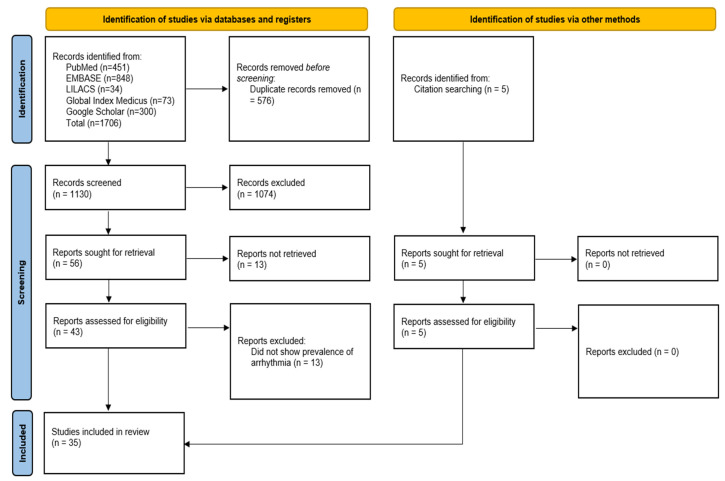
PRISMA flow diagram.

The database search identified 1706 records. After removal of 576 duplicates, 1130 unique records were screened by title and abstract, of which 1074 were excluded. Full texts were sought for 56 reports; 13 could not be retrieved. The remaining 43 full-text articles were assessed for eligibility, and 13 were excluded because they did not report the proportion of arrhythmias. An additional 5 studies were identified through citation searching, all were included. In total, 35 studies were retained for the quantitative synthesis [12,13,14,15,16,17,18,19,20,21,22,23,24,25,26,27,28,29,30,31,32,33,34,35,36,37,38,39,40,41,42,43,44,45,46]. Figure 1 shows the PRISMA flow diagram.

### 3.2. Proportion of Any Arrhythmia

Across all 35 studies, comprising 6948 patients with dengue, 1419 had at least one documented cardiac arrhythmia. Using a random-effects generalized linear mixed model (GLMM), the pooled proportion of any arrhythmia was 24.48% (95% CI 17.54–33.07), with very high between-study heterogeneity (Tau^2^ = 1.5426; Chi^2^ = 861.99, df = 34; *p* < 0.0001; I^2^ = 96.1%).

**Figure 2:**
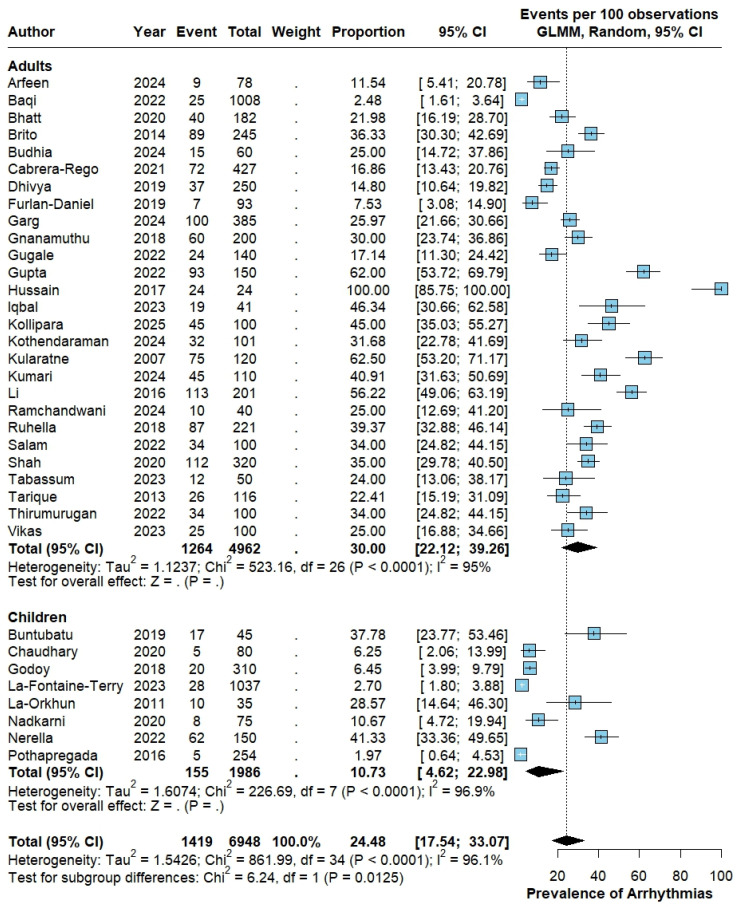
Pooled proportion of any cardiac arrhythmia in dengue. Forest plot of the proportion of any cardiac arrhythmia in patients with dengue, stratified by age group (adults vs. children), using a random-effects generalized linear mixed model (GLMM) with logit transformation; estimates are shown as events per 100 patients with 95% confidence intervals. The studies included in this analysis correspond to references [12,13,14,15,16,17,18,19,20,21,22,23,24,25,26,27,28,29,30,31,32,33,34,35,36,37,38,39,40,41,42,43,44,45,46].

When stratified by age group, adults (27 studies; 4962 patients) had a pooled proportion of 30% (95% CI, 22.12–39.26), with substantial heterogeneity (Tau^2^ = 1.1237; Chi^2^ = 523.16; df = 26; *p* < 0.0001; I^2^ = 95%). In children (8 studies; 1986 patients), the pooled proportion was 10.73% (95% CI 4.62–22.98), also with marked heterogeneity (Tau^2^ = 1.6074; Chi^2^ = 226.69, df = 7; *p* < 0.0001; I^2^ = 96.9%). A formal test for subgroup differences showed a statistically significant difference between adults and children (*p* = 0.0125; Figure 2).

### 3.3. Proportion of Specific Arrhythmia Subtypes

#### 3.3.1. Bradycardia

**Figure 3:**
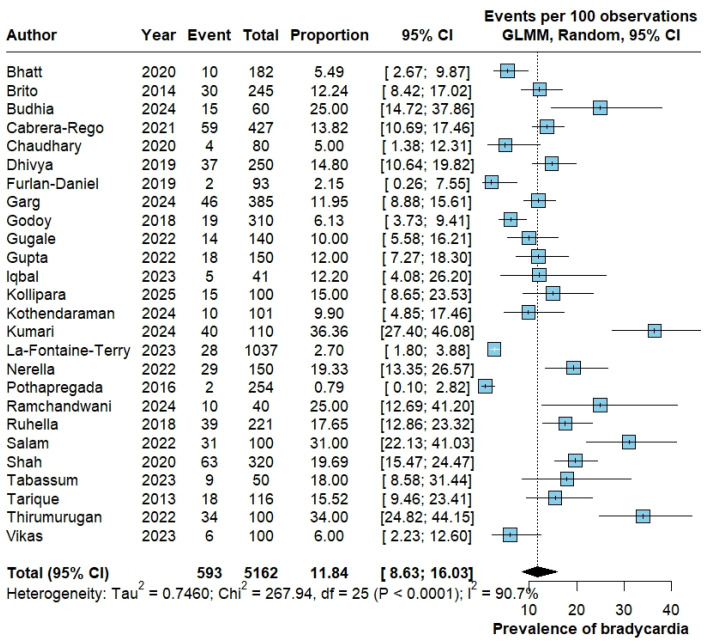
Pooled proportion of bradycardia in dengue. Forest plot showing study-specific and pooled proportion of bradycardia among patients with dengue, estimated with a random-effects generalized linear mixed model (GLMM) with logit transformation; results are expressed as events per 100 patients with corresponding 95% confidence intervals. The studies included in this analysis correspond to references [12,13,14,18,20,21,22,23,24,25,26,27,28,29,30,31,32,33,34,35,36,40,42,44,45,46].

For bradycardia, 593 events were reported among 5162 patients. The pooled proportion was 11.84% (95% CI 8.63–16.03), with considerable heterogeneity (Tau^2^ = 0.7460; Chi^2^ = 267.94, df = 25; *p* < 0.0001; I^2^ = 90.7%) (Figure 3).

#### 3.3.2. Tachycardia

**Figure 4:**
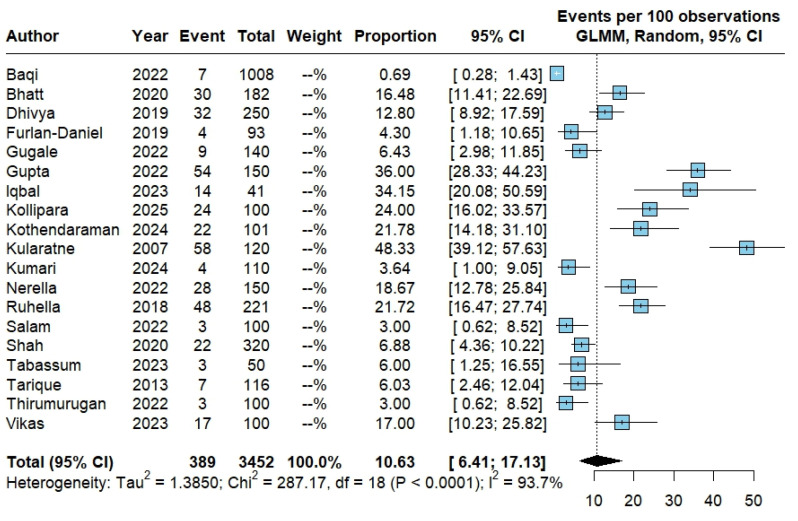
Pooled proportion of tachycardia in dengue. Forest plot showing study-specific and pooled proportion of tachycardia among patients with dengue, estimated using a random-effects generalized linear mixed model (GLMM) with logit transformation; results are expressed as events per 100 patients with corresponding 95% confidence intervals. The studies included in this analysis correspond to references [12,13,16,23,25,26,28,29,30,31,32,34,36,40,41,42,44,45,46].

Across studies reporting tachycardia, there were 389 events in 3452 patients, yielding a pooled proportion of 10.63% (95% CI 6.41–17.13) under a random-effects GLMM. Between-study heterogeneity was high (Tau^2^ = 1.3850; Chi^2^ = 287.17, df = 18; *p* < 0.0001; I^2^ = 93.7%) (Figure 4).

#### 3.3.3. Atrioventricular (AV) Block

**Figure 5:**
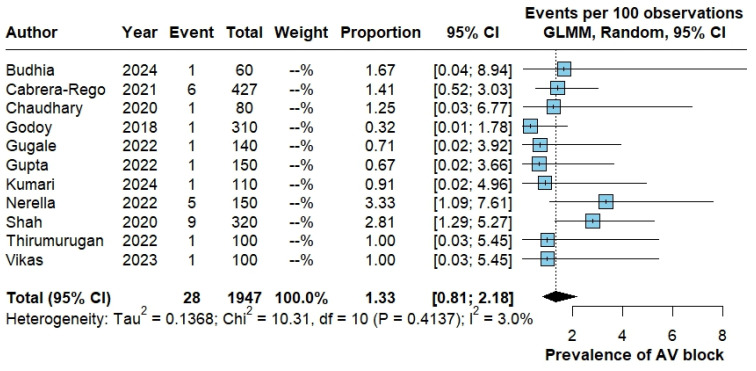
Pooled proportion of atrioventricular block in dengue. Forest plot showing study-specific and pooled proportion of atrioventricular (AV) block among patients with dengue, estimated using a random-effects generalized linear mixed model (GLMM) with logit transformation. Results are expressed as events per 100 patients with corresponding 95% confidence intervals. The studies included in this analysis correspond to references [12,21,24,25,26,27,28,32,35,42,44].

Eleven studies reported AV block, with 28 events among 1947 patients. The pooled proportion of AV block was 1.33% (95% CI 0.81–2.18). In contrast with other outcomes, heterogeneity was low (Tau^2^ = 0.1368; Chi^2^ = 10.31, df = 10; *p* = 0.41; I^2^ = 3.0%) (Figure 5).

#### 3.3.4. Other Arrhythmias

**Figure 6:**
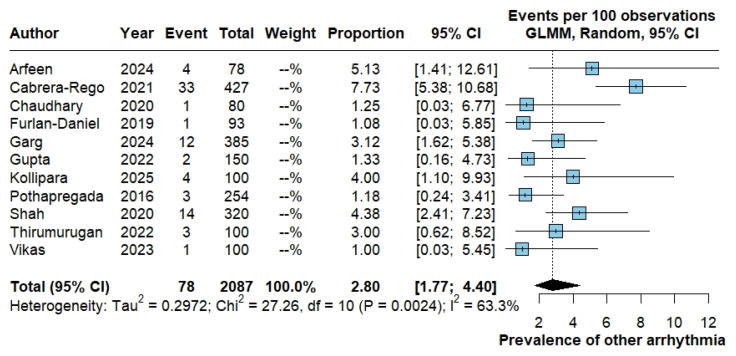
Pooled proportion of other arrhythmias in dengue. Forest plot showing study-specific and pooled proportion of other (non-specified) cardiac arrhythmias among patients with dengue, estimated using a random-effects generalized linear mixed model with logit transformation; results are expressed as events per 100 patients with corresponding 95% confidence intervals. The studies included in this analysis correspond to references [13,15,18,21,22,25,26,28,35,42,46].

For “other arrhythmias” (rare or unspecified rhythm disturbances not classified above), 78 events were documented in 2087 patients. The pooled proportion was 2.80% (95% CI 1.77–4.40), with moderate heterogeneity (Tau^2^ = 0.2972; Chi^2^ = 27.26, df = 10; *p* = 0.0024; I^2^ = 63.3%) (Figure 6).

### 3.4. Assessment of Publication Bias

The funnel plot of logit-transformed proportions against their standard errors for the main outcome (any arrhythmia) appeared approximately symmetrical (Supplement Figure S1). Egger’s regression test of the intercept showed no statistically significant evidence of funnel plot asymmetry (intercept −3.047; 95% CI −7.61 to 1.5; t = −1.312; *p* = 0.198), suggesting that small-study effects are unlikely to explain the observed findings fully.

### 3.5. Sensitivity Analysis and Influence Analyses

Influential-study sensitivity analyses based on the Baujat plot also yielded robust results (Supplement Figures S2 and S3). After excluding Baqi, the pooled proportion was 25.91% (95% CI 18.95–34.35; I^2^ = 95.1%); after excluding La-Fontaine-Terry, it was 25.87% (95% CI 18.88–34.34; I^2^ = 95.1%); and after excluding both studies, it was 27.45% (95% CI 20.58–35.59; I^2^ = 93.2%). These exclusions led to only modest changes in the pooled estimate, and the main conclusions remained unchanged (Supplement Figures S4–S6).

In a sensitivity analysis using an inverse-variance random-effects model with Freeman–Tukey double-arcsine transformation, the pooled proportion of any cardiac arrhythmia was 27.33% (95% CI 20.27–35.00), compared with 24.48% (95% CI 17.54–33.07) in the primary GLMM analysis. The estimates were broadly similar, with substantial overlap of confidence intervals, and therefore did not change the overall interpretation. Heterogeneity remained very high in both models (I^2^ = 96.1% and 97.9%, respectively) (Supplement Figures S7 and S8).

Exploratory subgroup analyses showed a lower pooled proportion of arrhythmias in studies from the Americas than in those from Asia, with a significant subgroup difference (*p* = 0.0176). Studies with laboratory-confirmed DENV infection also showed a lower pooled proportion than those without clear laboratory confirmation, although this difference was not statistically significant (*p* = 0.0853). Heterogeneity remained high across subgroups) (Supplement Figures S9 and S10).

## 4. Discussion

Dengue remains a major global public health problem, with substantial morbidity across endemic regions [3]. In this systematic review and meta-analysis (35 studies; 6948 patients), we found that cardiac arrhythmias were frequent during the index dengue illness (pooled proportion 23.32%), with a predominance of sinus bradycardia (11.84%) and sinus tachycardia (10.63%), whereas atrioventricular (AV) block was uncommon (1.33%). These findings support the concept that rhythm disturbances are a relevant—often under-recognized—component of dengue-associated cardiovascular involvement, particularly in settings where the clinical focus is appropriately centered on plasma leakage and shock [47].

Our pooled estimates align with, but are not identical to, prior syntheses and large hospital-based cohorts. A systematic review of cardiovascular sequelae in dengue (6773 patients) reported that ECG abnormalities were common overall, with sinus bradycardia and ST–T changes among the most frequently described findings [48]. Likewise, a focused review of electrocardiographic changes in dengue emphasized the predominance of sinus bradycardia and generally benign conduction disturbances, while acknowledging occasional clinically significant rhythm events [49]. In a large hospitalized cohort (*n* = 427), rhythm disorders were reported in ~17%, with sinus bradycardia as the leading abnormality and first-degree AV block observed at low frequency—figures that are directionally consistent with our finding that AV block is uncommon but non-negligible [35]. Differences in estimated proportion across studies are expected, given variability in case severity, monitoring intensity, and definitions of arrhythmia (rhythm disturbance vs. a physiologic response).

Mechanistically, dengue-associated rhythm disturbances can be conceptualized as the product of three overlapping pathways: (i) Hemodynamic stress from plasma leakage and shock physiology. Severe dengue is characterized by increased vascular permeability and intravascular volume depletion, which can provoke compensatory tachycardia and predispose to conduction instability due to ischemia and metabolic stress. Tissue Doppler data also suggest measurable cardiac functional impairment in severe dengue compared with non-severe cases, consistent with a cardio-hemodynamic contribution beyond simple “fever effects [50]”. (ii) Autonomic imbalance and relative bradycardia. Relative bradycardia has been described as a recognizable clinical feature of dengue and may account for a proportion of cases of sinus bradycardia, particularly around defervescence [7]. This reinforces that not all bradycardia implies myocarditis; some represents a reversible autonomic phenomenon in the course of acute viral illness [51]. (iii) Inflammatory myocardial involvement (myocarditis spectrum). Although uncommon, myocarditis is a recognized complication of dengue and may provide a substrate for more clinically significant conduction disturbances, including AV block [52].

Clinically, the dominance of sinus rate abnormalities should not be interpreted as uniformly benign. While many episodes are transient and resolve with defervescence and supportive care, rate and conduction changes may also mark clinically meaningful systemic stress (hypovolemia, electrolyte derangements) or, less commonly, myocardial involvement [52]. Therefore, the key clinical message is not simply that “arrhythmias occur,” but rather that arrhythmia detection can help identify patients who may benefit from closer hemodynamic assessment and targeted correction of reversible drivers.

The low pooled proportion of AV block (1.33%) should not lead to complacency. Even rare conduction disturbances can carry high clinical consequences when they occur in the context of hypotension, myocarditis, or electrolyte derangements. The clinical implication is that AV block—especially if progressive or symptomatic—should prompt systematic evaluation for reversible contributors (electrolytes, hypovolemia, medication effects) and consideration of myocarditis when clinically indicated, rather than being dismissed as an incidental ECG curiosity [53,54].

A central finding of this meta-analysis is the very high between-study heterogeneity for most rhythm outcomes. This is unsurprising in proportion meta-analyses and is likely explained by multiple interacting factors: (i) Monitoring strategy and timing. Studies relying on a single, non-standardized ECG are prone to miss transient events. (ii) Severity mix and clinical setting. Hospital cohorts often include patients with warning signs or severe dengue, in whom hemodynamic compromise is more common. (iii) Outcome definitions. The boundary between physiologic sinus tachycardia and a clinically relevant arrhythmia is not uniformly applied, and fever/hypovolemia can drive tachycardia independent of primary electrical instability. (iv) Baseline comorbidities. The reporting of underlying cardiovascular and systemic comorbidities was inconsistent across studies, limiting our ability to assess their influence in a standardized manner. Because preexisting conditions may affect both susceptibility to rhythm disturbances and the likelihood of cardiac evaluation, they represent a plausible source of residual confounding and between-study variability.

The higher pooled proportion of arrhythmias observed in adults than in children should be interpreted cautiously. Although adult age may contribute to cardiovascular vulnerability, dengue-related cardiovascular disorders have also been associated with male sex, thrombocytopenia, warning signs, and pre-existing cardiovascular disease. At the same time, pediatric studies show that cardiac involvement is also frequent in children and increases with disease severity [35]. In addition, broader prognostic meta-analyses do not support a simple age-only explanation, as childhood has been identified as a predictor of severe dengue overall [55]. Therefore, the adult–pediatric difference observed in our review is more likely to reflect differences in comorbidity burden, severity distribution, study setting, and monitoring intensity than age alone

Exploratory subgroup analyses suggested that part of the heterogeneity may be related to geographic setting and diagnostic confirmation. Studies from Asia showed a higher pooled proportion of arrhythmias than those from the Americas. Still, this finding should be interpreted cautiously because few studies came from the Americas, heterogeneity remained high within both strata, and La-Fontaine-Terry et al. included a mixed population spanning the Americas and Bangkok, making regional classification imperfect. Likewise, studies with laboratory-confirmed DENV infection showed a lower pooled proportion than those without clear laboratory confirmation, although this difference was not statistically significant. Overall, these findings suggest that region and diagnostic confirmation may contribute to between-study variability, but neither fully explains the substantial heterogeneity observed.

Strengths and limitations: This meta-analysis benefits from the comprehensive inclusion of observational evidence and the quantification of the proportion of subtype-specific arrhythmias. However, inference is constrained by (i) extreme heterogeneity, (ii) variability in monitoring modalities and timing, (iii) inconsistent arrhythmia definitions, and (iv) limited standardized reporting of disease severity and co-morbidities across studies. Future cohorts should pre-specify monitoring windows (e.g., febrile, critical, and convalescent phases), use standardized ECG/telemetry definitions, stratify by WHO severity classification, and report clinically meaningful endpoints (e.g., hemodynamic compromise, ICU admission, myocarditis, and post-acute cardiovascular outcomes).

Practical implications for care and surveillance: Our findings support a pragmatic monitoring strategy anchored in widely used dengue clinical frameworks. A reasonable approach is: (i) Baseline 12-lead ECG in hospitalized dengue patients, particularly adults, (ii) serial ECGs or telemetry for those with warning signs/severe dengue, abnormal baseline ECG, syncope/presyncope, chest pain, unexplained hypotension, significant electrolyte abnormalities, or suspected myocarditis (elevated troponin, new ventricular dysfunction). (iii) Interpret sinus tachycardia in context (fever, dehydration, anemia), avoiding over-classification as “arrhythmia” without supportive rhythm evidence.

Several limitations should be acknowledged when interpreting these findings. The evidence base was characterized by substantial heterogeneity in study design, clinical setting, disease severity, monitoring intensity, and arrhythmia definitions. Importantly, the pooled estimate was driven largely by sinus bradycardia and sinus tachycardia, findings that may in many cases reflect physiological responses to acute infection rather than clinically meaningful pathologic arrhythmias. Conversely, the frequent use of single ECG assessments may have missed transient or silent rhythm disturbances, particularly in studies without serial monitoring or telemetry. Because most included studies were hospital-based and often conducted in tertiary-care settings, the results may not be generalizable to broader dengue populations. Additional limitations include inconsistent laboratory confirmation and severity classification, incomplete reporting of key confounders, and the absence of individual-patient data, which precluded adjusted analyses and limited the interpretation of age-group differences. Estimates for less common rhythm disturbances were based on small numbers of events, and follow-up beyond the acute phase was rarely available. Taken together, these issues reduce the certainty of the evidence and support a cautious interpretation of the clinical significance of the reported rhythm abnormalities.

## 5. Conclusions

Dengue’s clinical impact remains highly relevant with multiple aspects to be specified regarding its frequency of occurrence and implications. In this systematic review and meta-analysis, rhythm abnormalities were reported in approximately 1 in 4 patients with dengue, with a higher proportion among adults than among children. However, this estimate should be interpreted cautiously, as most reported events were sinus bradycardia or sinus tachycardia, which may reflect transient physiological responses during acute illness rather than clinically significant pathologic arrhythmias. Although atrioventricular block was uncommon, it may still be clinically relevant in selected cases. The substantial between-study heterogeneity, likely related to differences in patient populations, disease severity, monitoring strategies, and arrhythmia definitions, limits the precision and generalizability of the pooled findings. Current evidence supports awareness of possible cardiac rhythm abnormalities in dengue, particularly in hospitalized patients, but does not justify broad conclusions about their overall clinical significance. Prospective studies using standardized ECG monitoring and uniform outcome definitions are needed to better characterize the frequency, timing, and prognostic relevance of dengue-associated rhythm disturbances.

## Data Availability

No new data were created or analyzed in this study.

## Supplementary Materials

The following supporting information can be downloaded at: https://www.mdpi.com/article/10.3390/pathogens15050497/s1, Table S1: Search strategies; Table S2: Characteristics of included studies; Table S3: Outcome characteristics; Table S4: Reporting for classification of dengue; Table S5: Laboratory test confirmation; Table S6: Summary of risk-of-bias assessment using the JBI checklist; Table S7: GRADE certainty of evidence; Figure S1: Funnel plot; Figure S2: Baujat plot; Figure S3: Baujat plot; Figure S4: Sensitivity analysis excluding Baqi et al.; Figure S5: Sensitivity analysis excluding La-Fontaine-Terry et al.; Figure S6: Sensitivity analysis excluding both Baqi et al. and La-Fontaine-Terry et al.; Figure S7: Pooled proportion of arrhythmias using the generalized linear mixed model; Figure S8: Pooled proportion of arrhythmias using the Freeman–Tukey method; Figure S9: Subgroup analysis by geographic region; Figure S10: Subgroup analysis by laboratory confirmation status.
