# Supplementary material for: Arrhythmias in Dengue: A Systematic Review and Meta-Analysis
Source: Pathogens. 2026 May 5;15(5):497. doi: 10.3390/pathogens15050497 (PMC13209691; doi:10.3390/pathogens15050497)
Supplement: Supplementary file 1 [file pathogens-15-00497-s001.zip › pathogens-4186798-supplementary.pdf]

Arrhythmias in dengue: A Systematic Review and Meta-Analysis.

Darío S. López-Delgado, Mathias S. Renteros-Ramirez,Joshua Emmanuel Arteaga-Bolaños, Harold E. Vásquez-Ucros,Kevin Alexander Burbano-Castro,Valentina Reina-Melo,Jessica Niebles-Blanco,Nancy Calzada-Gonzales Valmore Bermudez, Alfonso J Rodriguez-Morales.

SUPPLEMENTARY MATERIAL

Contents

Supplementary Table S1: Search strategies.. 2
Supplementary Table S2. Characteristics of included studies..................................... 4
Supplementary Table S3. Outcome Characteristics 13
Supplementary Table S4. Reporting for classification of dengue 27
Supplementary Table S5: Laboratory test confirmation.. 28
Supplementary Table S6. Summary of Risk of Bias Assessment (JBI Checklist)..... 29
Supplementary Table S7. GRADE Certainty of evidence 38
Supplementary Figure S1: Funnel Plot... 39
Sensitivity Analysis and Influence analisis 39
1. Supplementary Figures S2 and S3: Baujat plots 39
2. Supplementary Figures S4–S6: targeted influential-study sensitivity analysis, removing La-Fontaine-Terry and Baqi……………… … ….41
2. Supplementary Figures S7 and S8: Comparison between GLMM and Freeman-Tukey method 44
3. Supplementary Figures S9 and S10: Subgroup Analysis 46

Table S1. Search strategies

Database	Search date	Step	Search strategy	Results
PubMed/MEDLINE	May 28, 2025	#1	(Dengue[MeSH] OR dengue[tiab] OR "dengue fever"[tiab])	451
PubMed/MEDLINE	May 28, 2025	#2	("Arrhythmias, Cardiac"[MeSH] OR arrhythmi*[tiab] OR dysrhythmi*[tiab] OR tachycardia[tiab] OR bradycardia[tiab] OR "atrial fibrillation"[tiab] OR "atrial flutter"[tiab] OR "heart block"[tiab] OR "AV block"[tiab] OR "atrioventricular block"[tiab] OR "ventricular tachycardia"[tiab] OR "ventricular fibrillation"[tiab] OR "QT prolongation"[tiab] OR "long QT"[tiab] OR "rhythm abnormalit*"[tiab] OR "rhythm disturbance*"[tiab] OR cardiac[tiab])	—
PubMed/MEDLINE	May 28, 2025	#3	#1 AND #2	—
Global Index Medicus	May 28, 2025	#1	(MH:"Dengue" OR TW:(dengue OR "dengue fever"))	73
Global Index Medicus	May 28, 2025	#2	MH:"Arrhythmias, Cardiac" OR TW:(arrhythmi* OR dysrhythmi* OR tachycardia OR bradycardia OR "atrial fibrillation" OR "atrial flutter" OR "atrioventricular block" OR "AV block" OR "heart block" OR "ventricular tachycardia" OR "ventricular fibrillation" OR "QT prolongation" OR "long QT" OR "rhythm abnormalit*" OR "rhythm disturb*" OR cardiac)	—
Global Index Medicus	May 28, 2025	#3	#1 AND #2	—
EMBASE	May 28, 2025	#1	('dengue'/exp OR dengue:ti,ab OR "dengue fever":ti,ab)	848
EMBASE	May 28, 2025	#2	('heart arrhythmia'/exp OR arrhythmi*:ti,ab OR dysrhythmi*:ti,ab OR tachycardia:ti,ab OR bradycardia:ti,ab OR "atrial	—

Database	Search date	Step	Search strategy	Results
			fibrillation":ti,ab OR "atrial flutter":ti,ab OR "heart block":ti,ab OR "AV block":ti,ab OR "atrioventricular block":ti,ab OR "ventricular tachycardia":ti,ab OR "ventricular fibrillation":ti,ab OR "QT prolongation":ti,ab OR "long QT":ti,ab OR "rhythm abnormalit*":ti,ab OR "rhythm disturbance*":ti,ab OR cardiac:ti,ab)	
EMBASE	May 28, 2025	#3	#1 AND #2	—
LILACS	May 28, 2025	#1	(MH:"Dengue" OR TW:(dengue OR "dengue fever")) AND (MH:"Arrhythmias, Cardiac" OR TW:(arrhythmi* OR dysrhythmi* OR tachycardia OR bradycardia OR "atrial fibrillation" OR "atrial flutter" OR "heart block" OR "AV block" OR "atrioventricular block" OR "ventricular tachycardia" OR "ventricular fibrillation" OR "QT prolongation" OR "long QT" OR "rhythm abnormalit*" OR "rhythm disturbance*" OR cardiac))	34

Table S2. Characteristics of included studies

Author	Year	Country	Study design	Data source	Population	Average age /% of women
Arfeen et al. [15]	2024	India	Prospective observational study	Patna Medical College and Hospital, Patna; January–November 2023	78 patients with confirmed dengue (positive NS1 antigen and/or IgM antibodies); excluded: pre-existing cardiac disease, congenital heart disease, chronic cardiac medications	Mean age 35.4 ± 15.2 years; 66.7% male (52/78), 33.3% female (26/78)
Baqi et al. [41]	2022	Pakistan	Retrospective observational study	Electronic medical records of Aga Khan University Hospital, Karachi; Nov 2018–Nov 2019	Adults >18 years with confirmed dengue (NS1 or IgM positive); exclusions: prior cardiac disease, toxicity, autoimmune, renal failure, pregnancy, dual infections	Mean age not directly stated; 55.4% >35 years; 31.6% female
Bhatt et al. [36]	2020	India	Prospective observational study	Tertiary care center in Northern India, Sep–Nov 2018; confirmed dengue via NS1/IgM ELISA	Patients ≥14 years; exclusions: medications affecting HR/rhythm, pre-existing heart disease, electrolyte abnormalities, emergency ward <24h, pediatrics.	Mean age 30±12.6 years; 31% female
Budhia et al. [24]	2024	India	Prospective observational study	Department of General Medicine, Hi-Tech Medical College & Hospital, Bhubaneswar; Aug–Dec 2023	Adults 18–65 years with ≥2 dengue symptoms; dengue confirmed with NS1 Ag, IgG, IgM; exclusions: CKD, chronic hepatitis, negative serology.	Mean age 37.5 (SD 11.2); 52 males, 8 females
Buntubatu et al. [38]	2019	Indonesia	Prospective cohort study	Paediatric ward & PICU, Dr. Sardjito General Hospital, Yogyakarta; July 2015–May 2016	Children <18 years with confirmed dengue (WHO 2011 criteria, IgM/IgG or NS1 positive); exclusions: congenital/acquired cardiac disease, neuromuscular disorders	Median age 8 years (range 5 months–17 years); 70% male, 30% female

Cabrera-Rego et al. [35]	2020	Cuba	Observational, analytical, longitudinal, prospective epidemiological study	Hospital Docente Clínico-Quirúrgico Comandante Manuel Fajardo, Havana; Apr 2017–Apr 2018	Adults >18 years with classical dengue symptoms and positive IgM on day 6; exclusions: baseline cardiovascular disease (hypertensive heart disease, ischemic heart disease, non-infectious valvulopathies)	Age distribution provided by groups; no mean reported; % male 67.9%, % female 32.1%
Chaudhary et al. [21]	2020	India	Prospective observational study	Department of Pediatrics, Grant Government Medical College and Sir JJ Group of Hospitals, Mumbai	Children <18 years hospitalized with dengue confirmed by NS1 Ag and/or IgM; exclusions: congenital/acquired heart disease, drugs affecting HR/rhythm, electrolyte imbalance, pre-existing severe systemic illness	Mean ages: 6.83 y (no warning signs); 8.45 y (warning signs); 7.2 y (severe dengue); 66.25% male, 33.75% female
Dhivya et al. [23]	2019	India	Cross-sectional observational study	Department of Medicine, KIMS, Bengaluru; Nov 2012–Oct 2014	Adults ≥18 years with clinical dengue and NS1 or IgM positive, platelet count <100,000; exclusions: malaria, leptospirosis, enteric fever, scrub typhus	Mean age not reported; 57.6% female, 42.4% male
Espinosa Brito [33]	2014	Cuba	Prospective descriptive case series	Hospital General Universitario Dr. Gustavo Aldereguía Lima, Cienfuegos; June–August 1981	Adults ≥15 years with WHO 1975/1980 criteria for dengue hemorrhagic fever; 997 included; 1280 excluded (dengue without DHF)	Mean age 36.3 years; females 60.1%
Furlan-Daniel et al. [13]	2019	Brazil	Observational cross-sectional comparative study	Primary care services in Porto Ferreira, São Paulo; DENV outbreak Feb–Mar 2015	93 consecutive patients with confirmed non-complicated dengue (NS1 or ELISA IgM positive); controls: 93 healthy age- and sex-matched individuals	Mean age 71±14 years; 41% male, 59% female
Garg et al. [18]	2024	India	Prospective observational study	Tertiary care center, Western Uttar Pradesh; recruitment of confirmed dengue cases; institutional ethics committee approval obtained	296 confirmed dengue patients aged ≥12 years; diagnosis by NS1 antigen or IgM/IgG ELISA; exclusions: coronary artery disease, valvular heart disease, cardiomyopathy	Median age 29 years (IQR 21–38); 57.4% male, 42.6% female

Gnanamuthu et al. [17]	2018	India	Non-randomized prospective observational study	Government Rajaji Hospital, Madurai, Tamil Nadu; August–December 2017; bedside handheld 2D echocardiography and ECG performed	200 adult patients hospitalized with febrile thrombocytopenia (platelet <50,000/µL); includes dengue fever, DHF, and DSS; exclusions: platelet >50,000/µL, inherited platelet disorders, corticosteroid use, known cardiomyopathies	Mean age: males 24.12 yrs, females 28.32 yrs; overall mean age 29.43±13.71 yrs; 69% male (138/200), 31% female (62/200)
Godoy et al. [27]	2018	Paraguay	Observational, descriptive, longitudinal cohort study	Pediatric Emergency Department, Hospital General Pediátrico Niños de Acosta Ñu; March–September 2012	Children aged 1 month to 18 years with clinical and serological dengue diagnosis (IgM after day 7 or NS1 in first 3 days) who presented bradycardia; excluded congenital heart disease	Mean age 12.7 ± 2.8 years; predominance of males (16/20 = 80%)
Gugale et al. [44]	2022	India	Prospective observational study	Poona Hospital and Research Centre, Pune; June 2018–May 2019	140 adults ≥18 years with NS1-positive or IgM/IgG-positive dengue fever; exclusions: prior cardiac illness, ECG suggestive of old MI, meds affecting HR, concomitant infections	Mean age 41.6 ± 13.2 years; 62.1% male (87/140)
Gupta et al. [28]	2022	India	Prospective observational study	Tertiary care centre in Northern India; January–December 2019	150 hospitalized adults >18 years with confirmed dengue fever (NS1 or IgM positive by rapid test or ELISA); exclusions: pre-existing cardiac disease, electrolyte abnormalities, pregnancy, immunocompromised patients	Mean age 36.7 ±14.3 years; 66.7% male (100/150)
Hussain et al. [37]	2017	Pakistan	Retrospective review	Medical ICU, Ittefaq Hospital, Lahore; dengue epidemic July–October 2011	All adult dengue fever patients transferred to ICU; patients with hypotension classified into hypovolemic shock, cardiogenic shock, dengue hemorrhagic shock, and dengue leakage shock; myocarditis group consisted of	Mean age 32 years; 83.3% male (20/24), 12.5% female (3/24)

					patients with cardiogenic shock	
Iqbal et al. [34]	2023	Pakistan	Retrospective observational study	Department of Medicine, Sir Ganga Ram Hospital, Lahore; October–December 2022	64 hospitalized patients during dengue epidemic; 23 dengue fever (35.9%), 41 dengue hemorrhagic fever (64.1%); inclusion: fever 2–10 days, thrombocytopenia, leukopenia, capillary leak signs; exclusion: fever >10 days, normal platelets, other causes of fever	53.12% male (34/64), 46.87% female (30/64); age groups provided (12–45, 46–60, >60) but no mean age reported
Kothendaraman et al. [45]	2024	India	Hospital-based prospective observational study	Department of Medicine, Indira Gandhi Medical College and Research Institute, Pondicherry; adults >18 years with dengue (with or without warning signs)	101 adults ≥18 years with fever without focus and positive diagnostic test (NS1 or IgM) and classified as dengue with or without warning signs; exclusions: severe dengue, scrub typhus, enteric fever, pre-existing cardiac illness, rhythm disturbances	Sex distribution: 54.5% male (55/101), 45.5% female (46/101); age range 18–74 years with majority aged 20–29 years; mean not reported
Kularatne et al. [16]	2007	Sri Lanka	Prospective observational cohort study during dengue outbreak	General Hospital, Peradeniya, Sri Lanka; March–May 2005; adult general medical ward	120 serologically confirmed adult dengue fever patients; exclusions: DHF and DSS on admission, electrolyte/urea/creatinine abnormalities	Cardiac group median age 34 (range 12–76), 50 females (67%), 25 males (33%); Non-cardiac group median age 38 (range 15–68), 20 females (44%), 25 males (56%)
La-Fontaine-Terry et al. [14]	2023	Cuba	Descriptive, longitudinal, retrospective observational study	Provincial University Pediatric Hospital Dr. Eduardo Agramonte Piña, Camagüey; January–December 2021	28 adolescent patients (10–19 years old) diagnosed with dengue who presented bradycardia; inclusion: clinical diagnosis plus IgM >148 mg/dl from day 6; exclusion: congenital heart disease	57.1% male (16/28); 42.9% female (12/28); age groups: 10–14 years (35.6%), 15–19 years (64.2%)
La-Orkhun et al. [43]	2011	Thailand	Prospective observational	King Chulalongkorn Memorial Hospital, Bangkok;	35 previously healthy children aged 5–15 years with serologically or	Mean age 11.7 ± 2.3 years; 20 males

			Holter monitoring study	Pediatric ward; Holter monitoring during convalescent stage	PCR-confirmed dengue virus infection; monitored during convalescent stage ≥24 h after defervescence	(57.1%), 15 females (42.9%)
Li et al. [19]	2016	China	Large-sample observational study during dengue outbreak	Guangzhou 8th People's Hospital; August–October 2014; worst dengue outbreak in two decades	1782 hospitalized dengue patients; dengue confirmed by NS1, IgM/IgG ELISA, and/or RT-PCR; exclusions: history of myocarditis, MI, HF, CAD, valvular disease, congenital heart disease, autoimmune disease, renal failure, pregnancy	Age distribution not explicitly stated; no significant difference between myocarditis and non-myocarditis groups; sex distribution: myocarditis 58.71% male, 41.29% female
Nadkarni et al. [39]	2020	India	Hospital-based observational study	Department of Paediatrics, Gandhi Medical College and Hamidia Hospital, Bhopal; June 2015–August 2016	75 children (1 month–12 years) with confirmed dengue (NS1 or IgM positive) out of 206 suspected cases; exclusions: co-existing infections, pre-existing heart disease, pre-existing neurological disorder	Male:female ratio 1.8:1; mean age of cardiac subgroup 7.6 years (range 4–10 years); no overall mean age reported
Nerella et al. [32]	2022	India	Prospective observational hospital-based study	Tertiary care government hospital, Department of Pediatric Medicine, Kolkata; January 2017–June 2018	150 children aged 1 month–12 years seropositive for dengue (NS1 ELISA or IgM ELISA). Initial 170 enrolled; exclusions: 5 who received fluid bolus/RBC transfusion, 5 who did not consent, 10 without ECHO within 48 h; co-infections excluded (malaria, enteric fever, TB, pneumonia); no prior cardiac illness.	Age groups: 3 children <1 year; 55 children 1–6 years; 92 children 7–12 years. Sex distribution not explicitly listed; no mean age reported.
Pothapregada et al. [22]	2016	India	Retrospective observational study	Tertiary care hospital, Puducherry; August 1, 2012–January 31, 2015; WHO 2011 dengue criteria	254 children aged 0–12 years with confirmed dengue fever (NS1 or IgM/IgG positive); retrospective case record review; atypical manifestations analyzed	Mean age 6.9 ± 3.3 years; male:female ratio 1.2:1; age group 6–12 years most common (58.2%)

Ramchandwani et al. [20]	2024	India	Prospective observational study	Veer Surendra Sai Institute of Medical Sciences and Research (VIMSAR), Burla, Odisha; September 2023–January 2024	40 adult patients ≥18 years with suspected dengue confirmed by NS1, IgG or IgM positivity; exclusion: CKD, chronic hepatitis; monitored daily with CBC, LFT, RFT, ECG, X-ray, ultrasonography	Mean age 39.4 years (SD 12.3); 35 males (87.5%), 5 females (12.5%)
Ruhella et al. [29]	2018	India	Retrospective observational study	S.P. Medical College and Associated Groups Hospital, Bikaner, Rajasthan; June 1, 2016–May 30, 2017	Serologically confirmed dengue fever patients; exclusions: known pulmonary/cardiac/thyroid disease, age <18 or >60, medications affecting heart rate (β-agonists, β-blockers, digoxin, theophylline, calcium channel blockers)	127 males, 94 females (no mean age reported)
Kollipara et al. [46]	2018	India	Cross-sectional observational study	Department of General Medicine, Rajarajeswari Medical College and Hospital, Bangalore; Study period: March 2023 – March 2025	100 serologically confirmed dengue patients (NS1 antigen and/or IgM positive); Inclusion: age ≥18 years and consent; Exclusion: known cardiac disease, cardiac rhythm–affecting drugs, significant electrolyte disturbances.	Age groups shown in chart (page 1 image): 19–30 years largest group; followed by 31–40, 41–50, 51–60, and a smaller group 60–70 years. Exact mean not provided. Sex distribution from image: Males predominant; exact percentages not numerically stated.

Salam et al. [30]	2022	India	Prospective, cross-sectional study	Department of General Medicine, KVG Medical College, Sullia, Karnataka	100 serologically confirmed dengue patients, ages 15–60, evaluated with 12-lead ECG during febrile phase, ECG repeated every 24 hours for 5 days. Inclusion: high-grade fever 1–5 days, NS1/IgM/IgG positive. Exclusion: pulmonary/cardiac disease (RHD, DCM, conduction disorders, pacemaker), thyroid disease, age <15 or >60, medications affecting heart rate (β-agonists/antagonists, digoxin, theophylline), electrolyte imbalance (hypo/hyperkalemia, hypo/hypercalcemia).	Gender distribution: 80 males (80%), 20 females (20%). Mean age not reported.
Kumari et al. [12]	2024	India	Hospital-based retrospective study	Department of Medicine, Indira Gandhi Institute of Medical Sciences (IGIMS), Patna, Bihar; one-year study period	110 hospitalized dengue patients ≥13 years old with confirmed dengue serology (IgG/IgM/NS1 positive) meeting WHO criteria; exclusions: medications affecting HR/rhythm, preexisting heart disease, electrolyte abnormalities, mixed infections (malaria, leptospira), unwilling to consent	Mean age 35.15 ± 13.2 years (range 16–68); males 62%, females 38%; male mean age 33.9 ±13.5 (range 16–63); female mean age 37.05 ±12.5 (range 16–68)
Shah et al. [42]	2019	India	Prospective observational study	Kerala Institute of Medical Sciences, Trivandrum, Kerala; September 2016–August 2017	320 adult patients (>18 years) with confirmed dengue fever by serology; exclusions: preexisting coronary artery disease, heart failure, LV dysfunction, valvular disease, cardiomyopathy, cardiac arrhythmias, kidney dysfunction (creatinine >1.5 mg/dl).	Age distribution: 18–30 (26%), 31–50 (36.56%), 51–70 (32.81%), 71–80 (4.68%). Male 61.87% (198/320), Female 38.13% (122/320).

Tabassum et al. [40]	2023	Bangladesh	Cross-sectional observational study	Medicine Department, Dhaka Medical College Hospital; 1 October 2017–31 March 2018	50 hospitalized patients with dengue fever or dengue hemorrhagic fever; inclusion: high-grade fever with positive Dengue IgM, NS1 antigen, or RT-PCR; exclusion: electrolyte abnormalities, preexisting heart disease, drugs affecting heart rhythm	Mean age 33.08 ± 9.79 years; male 60% (30/50), female 40% (20/50); age groups: 15–20 (8%), 21–30 (38%), 31–40 (32%), 41–50 (18%), >50 (4%)
Tarique et al. [31]	2013	Pakistan	Cross-sectional analytical study	Department of Medicine, Mayo Hospital Lahore; September–November 2011	116 serologically confirmed dengue patients fulfilling WHO criteria for DF and DHF; clinical presentation, laboratory monitoring, chest radiograph and abdominal ultrasound (in DHF) recorded; ECG and cardiac enzymes checked within 24 hours of admission.	Mean age 32.88 ± 14.6 years; 94 males (81%) and 22 females (19%).
Thirumurugan et al. [25]	2022	India	Cross-sectional study	Government Villupuram Medical College and Hospital, Department of Cardiology; July–December 2021	100 consecutive patients aged ≥15 years with confirmed dengue serology (NS1 or IgM ELISA), fulfilling WHO criteria; exclusions: medications affecting heart rate/rhythm, pre-existing heart disease, electrolyte abnormalities, unwilling to consent	Mean age 45 years (range 15–85); 54% male (54/100), 46% female (46/100)
Vikas et al. [26]	2023	India	Cross-sectional study	Hospitals attached to Mysore Medical College and Research Institute, Mysore; November 2017–May 2019	100 confirmed dengue patients ≥18 years, NS1/IgM positive; WHO 2009 classification used; exclusions: medications affecting HR/rhythm, preexisting heart disease, electrolyte abnormalities, thyroid disorders	Most common age group 18–30 years; proportion males > females; exact male/female counts not provided

Table S3. Outcome Characteristics

Author	Year	N total / N % arrhytmia	Tipo de arrhytmia	Definition and measurement of arrhythmia	Main outcomes (overall)	Outcomes associated with presence of arrhythmia
Arfeen et al. [15]	2024	78 total; cardiac complications 19.2%; arrhythmias 4/78 (5.1%)	Arrhythmias (not subclassified), conduction defects	Arrhythmias identified by ECG at admission; further ECGs only if abnormalities present	Cardiac complications: myocarditis 7.7%, arrhythmias 5.1%, pericarditis 3.8%, heart failure 2.6%; reduced LVEF in 6, pericardial effusion in 3, wall motion abnormalities in 4; elevated troponin I in 10, elevated CK-MB in 8	Patients with cardiac complications had longer hospital stay (12.5 ± 4.2 vs 8.3 ± 2.1 days), higher ICU need (66.7% vs 12.7%), increased mortality (13.3% vs 1.6%); hemorrhagic manifestations (OR 3.75) and shock (OR 4.89) strong predictors
Baqi et al. [41]	2022	1008 total; myocarditis 4.16% (42/1008); ECG abnormalities in myocarditis: 59.5%	Sinus tachycardia, sinus bradycardia and atrial fibrillation within myocarditis; other ECG abnormalities (ST–T changes, conduction delays, poor R-wave progression) also reported.	ECG interpreted by cardiologist; abnormalities included tachy/bradyarrhythmia's, AV block, IVCD, AF, SVT, premature beats, VT/VF; ECG done when clinically indicated	Prevalence of myocarditis (4.16%); mortality 1.6%; myocarditis mortality 21.4%; associations of troponin, ECHO abnormalities, comorbidities with LOS and mortality	AF associated with higher mortality odds (OR 4.05); ECG abnormalities present in 59.5% of myocarditis cases; arrhythmias correlated with myocardial injury
Bhatt et al. [36]	2020	182 total; ECG abnormalities 24% (44/182); myocarditis 7.1% (13/182)	Sinus tachycardia and sinus bradycardia as rhythm disturbances; plus non-arrhythmic ECG changes such as ST-segment abnormalities.	Baseline ECG for all; biomarkers (Troponin I, NT-proBNP); ECG interpreted clinically; myocarditis diagnosed per ESC 2013 combining ECG, biomarkers, imaging	Myocarditis prevalence 7.1%; ECG abnormalities 24%; elevated biomarkers 14.8%; Echo abnormalities 6%; fluid overload more common in myocarditis; prolonged hospital stay; mortality 2.7% (all in	All myocarditis pts had ECG abnormalities; myocarditis associated with SOB, bleeding, high RR, fluid overload, longer LOS; arrhythmias part of diagnostic abnormalities

					myocarditis group)	
Espinosa Brito [33]	2014	997 total; ECG done in 245 pts; 36.7% (89/245) had ECG abnormalities; arrhythmias included within abnormalities	Sinus bradycardia (25%); myocarditis reported as a serious complication in some patients (including one fatal case), but no separate numeric breakdown for other arrhythmia subtypes.	ECG performed in 245 patients; arrhythmias identified from ECG tracings; some had follow-up ECG before discharge	Clinical characterization of DHF epidemic; symptoms, signs, hematologic findings, complications, ICU admissions, mortality (0.04%)	Bradycardia, AV block and conduction abnormalities transient and reversible; severe arrhythmias (VT/VF) occurred in one fatal case with splenic rupture and multiorgan failure
Budhia et al. [24]	2024	60 total; sinus bradycardia 25% (15/60)	Sinus tachycardia (11 children), sinus bradycardia (4), and combined sinus tachycardia + low-voltage QRS (2). No atrial fibrillation/flutter, supraventricular tachycardia, AV block, or ventricular tachycardia/fibrillation reported.	12-lead ECG performed on all patients; sinus bradycardia identified as ECG abnormality	Clinical symptoms (fever 100%, myalgia 90%, arthralgia 90%, GI symptoms 50%); CBC abnormalities (leukopenia 68%, thrombocytopenia 77%); LFT/RFT abnormalities (AST 93%, ALT 70%); pleural effusion 18%, ascites 12%; one death due to myocarditis	Sinus bradycardia present in 25%; associated with dengue illness; no subgroup analysis provided
Buntubatu et al. [38]	2019	50 total; ECG abnormalities 44% (22/45 ECGs); myocarditis 78% (39/50)	Sinus bradycardia; supraventricular and ventricular extrasystoles; first-degree AV block; atrial fibrillation; in addition, repolarization changes and pericarditis/myocarditis are described.	ECG interpreted by paediatric cardiology consultant; abnormalities included sinus tachy/bradycardia, low voltage QRS, ST-T alterations, prolonged QT, AV block, ventricular/atrial arrhythmia	Myocarditis prevalence 78%; CK elevated 56%; CK-MB 70%; Troponin I 24%; ECG abnormal 44%; myocarditis significantly more common in DSS (96%) vs DF (53%, p=0.003)	Arrhythmias/ECG abnormalities part of myocarditis criteria; myocarditis correlated with severity (highest in DSS); arrhythmias identified even in DF patients
Cabrera-Rego et al. [35]	2020	427 total; cardiovascular manifestations 19.7%	Sinus bradycardia; ST–T depression in precordial leads; first-degree AV block; RSR′	ECG tracings obtained from admission to discharge; arrhythmias	Cardiovascular manifestations 19.7%; pericarditis 1.6%; pericardial	Higher risk in males (RR 1.94), age 56–65 (RR 2.85) and >65 (RR 1.70),

		(84/427); sinus bradycardia 13.8%, atrial extrasystoles 4.9%, ventricular extrasystoles 4.0%, 1st-degree AV block 1.4%, atrial fibrillation 0.5%	pattern in V1–V2; other repolarization abnormalities. No supraventricular or ventricular tachyarrhythmias or high-grade AV block reported.	included sinus bradycardia, AV block, atrial/ventricular extrasystoles, atrial fibrillation, flutter, ventricular tachycardia; repolarization disorders defined as T-wave inversion/flattening or ST depression ≥0.5 mV in ≥2 contiguous leads	effusion 0.9%; myocarditis 0.2%; 75% occurred in first 7 days; 83.3% self-limited; advanced age, male sex, low platelet count, and dengue with warning signs significantly associated with cardiac involvement	low platelet count (RR 1.13), dengue with warning signs (RR 3.29); manifestations lasted 24–72 h in most patients
Chaudhary et al. [21]	2020	80 total; ECG abnormalities 21.2% (17/80)	Sinus bradycardia (14.8%); tachycardia (12.8%); ST-segment changes (0.8%); T-wave changes (4.0%); LVH (0.8%). No AF, SVT, VT or high-grade AV block explicitly described.	All patients received ECG; abnormalities included ST/T wave changes, sinus bradycardia, AV block. ECG performed at admission; interpreted clinically.	60% dengue without warning signs, 27.5% with warning signs, 12.5% severe dengue; cardiomegaly 8.75%; ECG abnormal 21.2%; CPK-MB and Troponin-I normal in all 80; ejection fraction similar across groups	ECG abnormalities not significantly associated with dengue severity; cardiac markers not elevated in any group; no correlation of arrhythmias with severity groups
Dhivya et al. [23]	2019	250 total; bradycardia 14.8%; tachycardia 12.8%; ECG normal in 66.5%	Sinus arrhythmias, predominantly sinus bradycardia (30 cases); AV conduction disturbances with first- and second-degree AV block; incomplete and complete right bundle branch block; left anterior fascicular block; ventricular repolarization abnormalities (ischaemic-like or diffuse).	ECG performed on all patients; arrhythmias defined through ECG abnormalities including bradycardia, tachycardia, ST/T changes, LVH	Clinical features: fever 100%, myalgia 97.2%, headache 94.4%; bleeding 11.6%; leukopenia 36%; SGOT↑ 59.6%, SGPT↑ 52.8%; ultrasound: hepatomegaly 56.4%, ascites 19.2%; outcomes: classical DF 86.8%, DHF 11.6%, DSS 0.8%, death 0.8%	Bradycardia noted in 14.8% but asymptomatic; no mortality associated; arrhythmia not analyzed separately regarding severity
Furlan-Daniel et al.	2019	93 dengue patients; sinus tachycardia 4%;	Sinus tachycardia, sinus bradycardia and atrial	12-lead ECG performed using digital system; QT	QTc significantly longer in dengue group vs controls	QTc prolongation seen in 5% of dengue patients; no

[13]		sinus bradycardia 2%; atrial fibrillation 1%; RBBB 6%; LBBB 1%; LVH 3%; ST-T changes 2%	fibrillation as rhythm disturbances; additional findings included right and left bundle branch block, left ventricular hypertrophy and ST–T changes, which are primarily conduction/repolarization abnormalities.	and QTc measured automatically; QTc calculated by Bazett formula; arrhythmias defined per ECG abnormalities	(407.4±35.3 vs 391.8±35.5 msec); 5% had abnormal QTc prolongation; no difference in QT dispersion; clinical features included malaise 97%, fever 96%, headache 88%, arthralgia 83%	ventricular repolarization dispersion abnormalities; ECG abnormalities may reflect mild myocarditis; no severe dengue cases included
Garg et al. [18]	2024	296 total; arrhythmia frequency: sinus bradycardia 17%; ST–T changes 10.8%; conduction disturbances 9%; arrhythmias overall reported across multiple categories	Sinus bradycardia; mild conduction disturbances; small group labelled simply as "arrhythmias"; ST–T changes are also reported but not counted as arrhythmias.	Cardiac manifestations assessed clinically and by electrocardiography; ECG abnormalities considered: sinus bradycardia, ST-T changes, conduction disturbances, arrhythmias per standard diagnostic criteria	Cardiac manifestations present in 111 of 296 patients; fever duration median 5 days; warning signs in 30.4%; shock in 5.4%; severe dengue in 8.1%; cardiac enzyme elevation in selected patients; laboratory abnormalities included leukopenia, thrombocytopenia, elevated hematocrit, liver dysfunction	Higher prevalence of cardiac involvement in severe dengue; conduction disturbances and repolarization changes noted more frequently in complicated dengue; bradycardia associated with autonomic dysfunction; ECG abnormalities observed more commonly in moderate-to-severe cases
Gnanamuthu et al. [17]	2018	200 total; sinus tachycardia 21 (10.5%); sinus bradycardia 4 (2%); 1st-degree AV block 2 (1%); RAD/RBBB 1 (0.5%); WNL ECG 172 (86%)	Sinus tachycardia; sinus bradycardia; first-degree atrioventricular block; right bundle branch block. No full n per subtype.	12-lead ECG obtained at admission; arrhythmias coded per ECG findings: tachycardia, bradycardia, AV block, conduction abnormalities; no troponin or advanced electrophysiologic testing	Cardiac symptoms in 20%; pleural effusion/ascites in 8.5%; pericardial effusion 12%; myocarditis 1% (global hypokinesia); cardiac chamber dilation 2%; abnormal valves 2%; right-sided effusion only; cardiac manifestations more common in DSS	Arrhythmias more common in DSS; tachycardia and bradycardia correlated with severe dengue presentations; myocarditis cases showed LV global hypokinesia; cardiac abnormalities influenced by BP and platelet count
Godoy et al.	2018	310 total dengue	Among the 20 bradycardia	Bradycardia defined as	Bradycardia onset median at	Most patients had severe

[27]		hospitalizations; 20/310 (6.4%) had bradycardia	cases, 19 had sinus bradycardia and 1 had first-degree AV block. All were ECG-documented.	HR below 25th percentile for age; ECG performed on all included patients; chest X-ray and echocardiogram performed when available	illness day 6 (range 2–8); pleural effusion on chest X-ray in 9/20; echo abnormalities: pericardial effusion (2/10), tricuspid regurgitation (1/10); mean hospital stay 7.1 ± 1.9 days; bradycardia persisted in 18/20 at discharge; resolved in first week follow-up in most cases	dengue; bradycardia appeared during hospitalization in 11/20; persisted at discharge despite clinical improvement; AV block resolved spontaneously; all patients recovered without cardiovascular sequelae
Gugale et al. [44]	2022	140 total; ECG abnormalities in 30 patients (21.4%)	Sinus bradycardia (14 patients; 10%), sinus tachycardia (9; 6.4%), and atrioventricular block (1; 0.7%); non-specific ST–T changes and right bundle branch block also reported but not counted as arrhythmias here.	Three serial ECGs performed (day 1, day 3, day 7/discharge); abnormalities defined as sinus tachy/bradycardia, ST-T changes, RBBB, AV block; 2D echo assessed systolic/diastolic function, EF<45%, pericardial effusion	Cardiac manifestations 21.4% (30/140); ECG abnormalities 21.4%; echo abnormalities 7% (systolic dysfunction 7 pts, EF<45% in 5, diastolic dysfunction 1); dengue severity distribution: DF 33.6%, DHF 49.3%, DSS 17.1%; 1 death reported	ECG abnormality rates increased with severity (DF 17%, DHF 20.3%, DSS 33.3%) but not statistically significant; patients with echo abnormalities also had ECG abnormalities; no significant association between cardiac abnormalities and age, gender, platelet count, or type of dengue
Gupta et al. [28]	2022	150 total; ECG abnormal in 64.6%; sinus tachycardia 36%; ST-T changes 25.3%; low-voltage complexes 20.6%; sinus bradycardia 12%; second-degree AV block 0.7%	Sinus tachycardia and bradycardia; occasional ventricular extrasystoles; one case of second-degree AV block; multiple non-rhythmic ECG abnormalities (ST–T changes, low voltage, poor R progression, wide QRS).	Electrocardiography performed on all patients; abnormalities included tachy/bradyarrhythmias, conduction defects, ST-T changes; classified per ESC 2013 myocarditis diagnostic criteria	Cardiac involvement suspected in 27.3% based on ESC criteria and strain imaging; biomarkers elevated: CPK-MB 28.6%, Troponin-T 20.6%; myocarditis more common in males; severe dengue associated with lower strain	Myocarditis group had higher hematocrit (53%); elevated cardiac enzymes significantly associated; ECG abnormalities common in myocarditis; reduced longitudinal and circumferential strain in severe dengue;

					values; LVEF ≤50% in 3.3%	myocarditis patients had shorter hospital stay (4.1 vs 5.5 days)
Hussain et al. [37]	2017	Among 128 ICU dengue admissions, 24 (19.67%) diagnosed as myocarditis; arrhythmias in myocarditis group: sinus tachycardia 87.5%, atrial fibrillation 8.3%, ventricular tachycardia 4.1%	Sinus tachycardia, atrial fibrillation, and ventricular tachycardia in patients with dengue myocarditis. No bradyarrhythmias or high-grade AV block are reported.	Electrocardiography performed in all myocarditis patients; ECG abnormalities defined as sinus tachycardia, atrial fibrillation, ventricular tachycardia; echocardiography used to confirm myocardial involvement (global hypokinesia, EF <40%, chamber dilatation)	Myocarditis occurred in 24/122 (19.67%) patients with shock; clinical features included 100% SOB, palpitations, fatigue, hypotension; elevated Troponin T (6.75 µg/L) and CK (950 U/L) in all 24; echo: global hypokinesia, EF<40%, biventricular dilation in 100%; mortality 83.3% (20/24)	Arrhythmias were part of myocarditis presentation; ventricular tachycardia associated with critical instability; arrhythmias occurred alongside high biomarker elevation and severe myocardial depression; myocarditis identified as a major contributor to mortality compared with other shock types
Iqbal et al. [34]	2023	64 total; among DHF critical phase group: tachycardia 34.14% (14/41), bradycardia 12% (5/41); overall ECG normal 53.86%, abnormal 46.14%	Tachycardia in 14/41 (34.1%) and bradycardia in 5/41 (12.2%); not further subclassified (e.g., sinus vs other supraventricular rhythms).	ECG interpreted for tachycardia and bradycardia during critical phase; echocardiography performed only in cases with abnormal ECG; capillary leak confirmed by chest X-ray, ultrasound, hematocrit, capillary refill	Clinical profile: fever 100%, bleeding 8%, tachycardia 34.14%, bradycardia 12%, diarrhea 14%, abdominal pain 20%, cough 3%; labs: thrombocytopenia 76%, HCT >20% in 35.93%, WBC <4000 in 75%	Arrhythmias mostly benign and self-limiting; tachycardia and bradycardia occurred during critical phase with capillary leak; bradycardia requires close monitoring due to hypotension risk; mechanism may involve altered autonomic tone, electrolyte imbalance, subclinical myocarditis
Kothendaraman et al. [45]	2024	101 total; ECG abnormalities uncommon; sinus tachycardia 21.8%	Sinus tachycardia and sinus bradycardia were the main rhythm abnormalities. Other ECG changes (not counted as	12-lead ECG recorded at 25 mm/s and 10 mm/mV using CARDIART 6108T; ECG	ECG abnormalities rare overall; most common abnormality sinus tachycardia; diastolic	Arrhythmias mainly sinus tachycardia and sinus bradycardia; no association found between

		(22/101); sinus bradycardia 9.9% (10/101); ST elevation 2%; T-wave inversions 2–3% depending on lead distribution; low voltage complexes 2%	arrhythmias here) included low-voltage QRS complexes, ST-segment elevation, and T-wave inversion in precordial leads.	abnormalities defined as tachycardia, bradycardia, ST elevation, T-wave inversion, and low-voltage QRS; interpreted in association with hypovolemia assessment	dysfunction present in 20.8%; hypokinesia 5%; pericardial effusion 1%; 31/101 had abnormal IVCCI; no agreement between IVCCI and clinical grading or diastolic dysfunction (kappa≈0.05–0.09); all patients survived with no morbidity	arrhythmias and dengue clinical severity; arrhythmias considered reflective of volume status rather than myocardial injury
Kularatne et al. [16]	2007	120 total; 75/120 (62.5%) had ECG abnormalities; among cardiac group: 58/75 (77%) had tachycardia or bradycardia; bradycardia prominent especially in secondary dengue	Sinus tachycardia and sinus bradycardia as the principal rhythm disturbances; additional ECG abnormalities included widespread T-wave inversion, ST-segment depression, right bundle branch block and occasional ST elevation, treated as conduction/repolarization abnormalities rather than separate rhythm diagnoses.	12-lead ECG daily; tachycardia defined as HR>100, bradycardia HR<60; ECG criteria for cardiac involvement: T inversion ≥2mm in >9 leads, ST depression ≥1mm in >3 leads, bundle branch block reversible with fever resolution	75/120 (62.5%) had cardiac abnormalities; 17/75 (23%) developed hypotension; 60/75 (80%) had hypotension or tachy-brady arrhythmias; 5 troponin-positive cases; 2 progressed to cardiogenic shock; DEN-3 detected in 3 patients; all recovered	Cardiac group had more fatigue, dyspnea, low SaO2 (85%), chest pain; bradycardia more common in secondary dengue; arrhythmias appeared early (day 3); echocardiography showed LVEF<55% in some; arrhythmias correlated with hypotension and myocardial dysfunction
La-Fontaine-Terry et al. [14]	2023	1037 dengue hospitalizations; 28/1037 (2.70%) developed bradycardia (100% sinus bradycardia)	Sinus bradycardia exclusively; no other arrhythmia subtypes are described.	Bradycardia defined as heart rate <60 bpm for adolescents; diagnosis by clinical assessment and ECG; weekly follow-up until resolution	Bradycardia onset: at admission 25%, during hospitalization 42.8%, at discharge 32.1%; pleural effusion in 46.4%; no pericardial effusion in most cases; duration: 6–10 days in 50%, >10 days in 32.1%	Bradycardia more frequent in dengue without warning signs (53.5%); occurred mainly during recovery phase; persisted at discharge in many cases; all patients recovered without cardiovascular sequelae
La-Orkhun et al. [43]	2011	35 total; 10/35 (29%) had cardiac rhythm	Sinus pauses (sinoatrial exit block), first-degree AV block,	18–24h overnight Holter monitoring; arrhythmias	29% had rhythm abnormalities; abnormalities	Arrhythmias benign and transient; not correlated

		abnormalities (excluding sinus bradycardia)	Mobitz type I second-degree AV block (Wenckebach), premature ventricular complexes (PVC), and premature atrial complexes (PAC). No SVT, VT, or high-grade AV block beyond Mobitz I.	identified descriptively: sinus pause, PAC/PVC, first-degree AV block, Mobitz I; HRV measured via 5-minute time and frequency domain analysis; ECG tracings reviewed manually for accuracy	benign and asymptomatic; no relationship between arrhythmia incidence and disease severity (DF, DHF, DSS); HRV showed no significant difference between convalescence and follow-up; indicates arrhythmias not mediated by parasympathetic overactivity	with dengue severity; Wenckebach phenomena possibly functional; no sustained tachyarrhythmias; ectopic beats and AV block episodes asymptomatic; autonomic imbalance not demonstrated by HRV metrics
Li et al. [19]	2016	1782 total; 201 myocarditis cases (11.28%); ECG abnormal in 153/201; arrhythmias on admission in myocarditis group 113/201 (56.22%)	Supraventricular tachycardia; atrial fibrillation; atrial premature beats; ventricular premature beats; atrioventricular block (I–III); intraventricular conduction block; ST–T changes.	12-lead ECG performed on admission and repeated if symptoms developed; ECG abnormalities defined per ESC 2013 criteria including arrhythmias, conduction defects, ST/T changes, low voltage, Q-wave abnormalities; UCG and cardiac enzyme tests also used	Myocarditis prevalence 11.28%; higher occurrence in NSD(WS+)/SD (46.66%) vs NSD(WS–) (9.72%); myocarditis patients had longer hospital stay (7.17±4.64 vs 5.98±2.69 days); higher AST, AST/ALT ratio; lower hemoglobin and hematocrit; more shock cases; ECG abnormality most sensitive diagnostic method	Arrhythmias common in myocarditis: 56.22% on admission; supraventricular tachycardia (14.29%) and atrial fibrillation (25.71%) more frequent in severe dengue; arrhythmias associated with increased clinical symptoms and higher rates of heart failure indicators; no difference in arrhythmias between groups at discharge
Nadkarni et al. [39]	2020	75 total; 10/75 (13.33%) had cardiac manifestations; sinus bradycardia in 5/10 (50%), tachycardia in 3/10 (30%), signs of CCF 3/10 (30%), hypotension/shock in	Among 10 children with cardiac manifestations: sinus bradycardia in 5, tachycardia (with signs of congestive cardiac failure) in 3; 2 had shock. No higher-grade AV block, atrial fibrillation/flutter, or ventricular arrhythmias	ECG performed in all 10 cardiac cases; arrhythmias defined as sinus bradycardia and sinus tachycardia; echocardiography post-stabilization; CK-MB measured and elevated in	Cardiac manifestations in 13.33%; 100% thrombocytopenia (<100,000/mm3); CK-MB markedly elevated (mean 306.9±246.95); tachycardia with CCF in 3 cases improved in 3 days; one	Bradycardia resolved upon recovery; no deaths in cardiac group; arrhythmias associated with thrombocytopenia and elevated CK-MB; hypotension/shock in 20% required

		2/10 (20%)	reported.	all 10	patient had mild pericardial effusion; no LV dysfunction/global hypokinesia	dopamine/milrinone; cardiac abnormalities considered mild and reversible
Nerella et al. [32]	2022	150 total; 78/150 (52%) had ECG abnormalities. Rhythm abnormalities: sinus tachycardia 28/150 (18.6%); sinus bradycardia 29/150 (19.3%); first-degree AV block 4/150 (2.6%); second-degree AV block 1/150 (0.6%). Severe dengue subgroup showed highest arrhythmia burden (Table 1).	Sinus tachycardia and bradycardia; first- and second-degree AV block; other ECG findings (PR prolongation, ST depression, low voltage) are reported separately as non-rhythmic abnormalities.	12-lead ECG (Clarity ECG 100-C; 25 mm/s). Parameters: heart rate, rhythm, voltage, PR interval, ST segment. Age-based PR prolongation cut-offs defined. Echocardiography within 48 h by pediatric cardiologist: EF by Teichholz method; diastolic function by E/A ratio; IVC collapsibility measured as volume indicator. Disease classified per 2015 National Dengue Guidelines.	ECG abnormalities: 52% overall. ECHO abnormalities: 46% (70/150). Systolic dysfunction: EF <55% in 63/150 (42%); moderate-to-severe EF reduction more common in severe dengue (Table 3). Diastolic dysfunction: E/A<1 in 15/150 (10%). Pericardial effusion: 11/150 (7.3%). IVC collapsibility >50% strongly associated with severe dengue (63.6%). Mortality 1.3% (2 cases).	Arrhythmias more common with increasing severity: sinus bradycardia in 50% of severe dengue; tachycardia similarly increased. Low-voltage QRS and ST-segment depression highest in severe dengue. Significant associations: low EF with disease severity (P=0.001), abnormal IVC collapsibility with severity (P=0.001). ECG and ECHO abnormalities reflect myocardial involvement and correlate with hemodynamic instability.
Pothapregada et al. [22]	2016	254 total; myocarditis 5 (1.9%); sinus bradycardia 2 (0.8%); paroxysmal supraventricular tachycardia 3 (1.2%); ectopic ventricular beats reported	Paroxysmal supraventricular tachycardia (3 cases); sinus bradycardia (2 cases); ectopic ventricular beats (number not specified); myocarditis (5 cases) and pericardial effusion (3 cases) as part of cardiac involvement.	ECG recorded when cardiac involvement suspected; sinus bradycardia and PSVT identified clinically and by ECG; myocarditis diagnosed via echocardiography showing global	Atypical manifestations in 41.7%; myocarditis in 5 children (global hypokinesia in 3, diastolic dysfunction in 2); pericardial effusion in 3; shock common (37.4%); refractory shock in 6; multiorgan involvement in 16; ARDS 4; AKI 6;	PSVT: one unstable case reverted with adenosine; bradycardia associated with shock; myocarditis patients required inotropes but normalized on discharge; arrhythmias were transient and responsive to supportive

				hypokinesia or diastolic dysfunction	neurological manifestations 28; 6 deaths (2.4%)	care
Ramchandwani et al. [20]	2024	40 total; sinus bradycardia in 10 patients (25%)	Sinus bradycardia only; no other arrhythmia subtypes (AF, SVT, VT, high-grade AV block) specified.	12-lead ECG performed in all patients; sinus bradycardia identified as HR <60 bpm; ECG part of routine assessment along with imaging	Clinical symptoms: fever 100%, myalgia/headache/arthralgia 90%, vomiting/diarrhea 50%, SOB 17.5%, abdominal swelling 10%; CBC abnormalities: leukopenia 67.5%, thrombocytopenia 77.5%, lymphocytosis 37.5%, eosinopenia 57.5%; LFT/RFT: AST ↑92.5%, ALT ↑70%, bilirubin ↑10%, proteins ↓67.5%, calcium ↓82.5%, phosphate ↓33.25%; imaging: pleural effusion 22.5%, ascites 12.5%; 1 death due to myocarditis (2.5%)	Sinus bradycardia occurred in 25% and considered part of dengue-related physiological response; no detailed subgroup correlation provided; myocarditis occurred in one fatal case
Ruhella et al. [29]	2018	221 total; 48 (21.7%) sinus tachycardia; 39 (17.6%) sinus bradycardia; 12 (5.4%) additional ECG changes	Sinus tachycardia; sinus bradycardia; nonspecific ST–T changes (11 cases); right bundle branch block (1 case); all reversible at discharge	12-lead ECG at admission and discharge; arrhythmias defined as sinus tachycardia or bradycardia; ST–T changes and RBBB also documented as ECG abnormalities	134 normal sinus rhythm; ECG changes included sinus tachycardia, bradycardia, nonspecific ST–T changes, reversible RBBB; all abnormalities resolved by discharge	Bradycardia highlighted as the most prominent cardiac manifestation; all arrhythmias reversible; ECG suggested as early diagnostic clue in dengue fever
Kollipara et al. [46]	2018	221 total; 48 (21.7%) sinus tachycardia; 39	Sinus tachycardia; sinus bradycardia; nonspecific ST–T	12-lead ECG at admission and	134 normal sinus rhythm; ECG changes included sinus	Bradycardia highlighted as the most prominent

		(17.6%) sinus bradycardia; 12 (5.4%) additional ECG changes	changes (11 cases); right bundle branch block (1 case); all reversible at discharge	discharge; arrhythmias defined as sinus tachycardia or bradycardia; ST–T changes and RBBB also documented as ECG abnormalities	tachycardia, bradycardia, nonspecific ST–T changes, reversible RBBB; all abnormalities resolved by discharge	cardiac manifestation; all arrhythmias reversible; ECG suggested as early diagnostic clue in dengue fever
Salam et al. [30]	2022	100 total; 49/100 (49%) had abnormal ECG findings. Among DF: 27 bradycardia, 2 tachycardia, 10 ST–T changes, 2 new RBBB. Among DHF: 5 bradycardia, 2 ST–T changes, 1 tachycardia.	Sinus bradycardia; sinus tachycardia; non-specific ST–T changes; new onset right bundle branch block (RBBB). No ectopics, no atrioventricular block.	12-lead ECG performed at admission and repeated every 24 hours for 5 days during febrile phase. Abnormalities assessed: heart rate (bradycardia, tachycardia), ST–T changes, conduction defects (RBBB).	49% abnormal ECG; sinus bradycardia most common (31%); ST–T changes 12%; sinus tachycardia 3%; RBBB 2%. No myocarditis, myocardial infarction, cardiac failure, or deaths reported. All ECG changes were reversible by discharge.	Bradycardia predominated in both DF and DHF; arrhythmias were transient and resolved spontaneously. ECG abnormalities were more frequent in DHF than DF, but difference not statistically significant. No patient with arrhythmia developed chest pain or clinical cardiac complications.
Kumari et al. [12]	2024	110 total; ECG abnormalities on admission 43.64%; by rhythm: 59.09% normal sinus rhythm, 36.4% sinus bradycardia, 3.6% sinus tachycardia, 10.9% NSST-T changes; 0.9% first-degree AV block; 1.8% QTc prolongation;	Sinus bradycardia, sinus tachycardia, first-degree AV block, right bundle branch block; NSST-T changes and QTc prolongation are common co-existing non-rhythmic ECG abnormalities.	ECG done on admission (afebrile), repeated on day 3 and at discharge; ECG parameters: HR, PR interval, ST-T alterations, QRS amplitude, QT interval; arrhythmias defined per standard ECG criteria; cardiac enzymes (Troponin I, CK-MB) and	Cardiac symptoms present in 60.09%; ECG abnormalities transient—100% normal at discharge; echocardiography: pericardial effusion 0.91%, systolic dysfunction 1.82%, diastolic dysfunction 2.73%; Troponin I positive in 6/8 DSS (75%), 7/26 DHF (26.9%), 0/76 DF; CK-MB	Sinus bradycardia predominant and transient; arrhythmias more common in DHF and DSS; positive troponin and CK-MB strongly associated with disease severity; systolic/diastolic dysfunction seen mostly in DSS; ECG and cardiac

		RBBB reported in some cases		echocardiography used to assess myocardial involvement	>25 IU in 87.5% DSS, 42.3% DHF, 39.5% DF; no mortality	enzyme abnormalities correlated significantly with severity (p<0.05)
Shah et al. [42]	2019	320 total; 112/320 (35%) had cardiac involvement. ECG abnormalities in 112 (35%); sinus bradycardia 19.7% (63/320); sinus tachycardia 6.9%; 1° AV block 1.9%; 2° AV block 0.3%; complete heart block 0.6%; atrial fibrillation 3.75%; ventricular tachycardia 0.6%.	Sinus bradycardia and tachycardia; AV block (first, second, complete); atrial fibrillation; ventricular tachycardia; plus ST-segment changes and systolic dysfunction.	12-lead ECG used to detect rate and rhythm abnormalities, heart block, ST/T changes; cardiac involvement criteria included symptoms (chest pain, dyspnea, palpitations, hypotension), ECG abnormalities, ECHO abnormalities (LVEF <50%, RWMA, pericardial effusion), and elevated biomarkers (hs-Troponin T >25 pg/ml, CK-MB >5 mg/ml, NT-ProBNP age-adjusted thresholds).	Cardiac involvement in 43.75% of dengue cases; ECG abnormalities 35%; ECHO abnormalities 21.9%; serum cardiac markers elevated in 31.6%; sinus bradycardia most common ECG abnormality. 70 patients had LVEF ≤50%; RWMA in 10.93%; segmental hypokinesia in 1.25%. 14 deaths (4.37%), all had ECG, ECHO, and biomarker abnormalities. Normal ECG, ECHO, and biomarkers had 100% negative predictive value for mortality.	Sinus bradycardia generally benign when not accompanied by elevated biomarkers. High-risk patterns included low LVEF (<40%), elevated troponin, and presence of RWMA. Mortality associated with complete heart block, ST-T abnormalities, VT, and severe myocardial injury. Patients with only sinus tachycardia or atrial fibrillation had no deaths.
Tabassum et al. [40]	2023	50 total; 16/50 (32%) had ECG abnormalities; bradycardia 9/50 (18%), tachycardia 3/50 (6%), T-wave inversion 2/50 (4%), ST elevation 1/50 (2%), ST depression 1/50 (2%)	Among 50 patients, 34 (68%) had normal ECG; 16 (32%) had abnormal findings. Abnormalities included bradycardia in 9 (18%), tachycardia in 3 (6%), T-wave inversion in 2 (4%), ST elevation in 1 (2%), and ST depression in 1 (2%). For this review, arrhythmias considered are bradycardia and tachycardia.	Standard 12-lead ECG performed on day 3 of illness and on discharge; ECG evaluated for heart rate, rhythm, P wave, T wave, ST segment, QRS complex; abnormal findings recorded and compared between DF and DHF	ECG normal in 68% (34/50); abnormal in 32% (16/50); bradycardia most common abnormality (>50% of abnormal ECGs; p<0.01). No significant correlation between ECG changes and disease severity (p=0.725). All transient ECG changes normalized at discharge; one patient with ST elevation diagnosed with acute MI. No	Bradycardia predominant and asymptomatic in all cases; tachycardia associated with palpitations; ST elevation case symptomatic with chest pain/dyspnea and diagnosed as MI; arrhythmias frequently asymptomatic; transient abnormalities resolved by discharge

					deaths.	
Tarique et al. [31]	2013	116 total; 78 patients had normal ECG (67.2%). Abnormal ECG findings included: tachycardia, bradycardia, supraventricular tachycardia, left bundle branch block, ST depression, poor R-wave progression. DF group (n=61): 13 bradycardia, 2 tachycardia, 3 ST depression, 1 new LBBB, 1 SVT, 1 poor R-wave progression. DHF group (n=55): 5 bradycardia, 5 tachycardia, 2 LBBB, 4 ST depression, 1 poor R-wave progression.	Sinus bradycardia; sinus tachycardia; supraventricular tachycardia; left bundle branch block; ST depression; poor R-wave progression.	12-lead ECG performed within 24 hours of admission; repeated monitoring for abnormalities including rate, rhythm, ST–T changes, conduction defects. Cardiac enzymes also tested.	ECG abnormalities occurred in both DF and DHF. Common abnormalities were bradycardia and ST depression. No deaths; none developed angina, myocardial infarction, or cardiac failure. Clinical features included palpitations (8.6%) and shortness of breath (8.6%).	Bradycardia showed borderline association with DHF (p=0.069). Symptoms such as dyspnea and palpitations were not significantly correlated with ECG abnormalities. All arrhythmias were transient, and all patients were discharged without cardiac complications.
Thirumurugan et al. [25]	2022	100 total; sinus bradycardia in 34%; sinus tachycardia in 3%; ventricular bigeminy 1%; ventricular trigeminy 1%; ventricular tachycardia 1%; AV	Sinus bradycardia (34 patients); sinus tachycardia (3); ventricular bigeminy, ventricular trigeminy and ventricular tachycardia (1 each); AV dissociation with sinus node dysfunction (1); ST–T changes in 11 patients. All	12-lead ECG for all patients; rhythm abnormalities identified as bradycardia (<60/min), tachycardia (>100/min), ventricular arrhythmias (bigeminy, trigeminy, VT), AV	Most common cardiac abnormality was sinus bradycardia (34%); ventricular arrhythmias and AV dissociation were transient, resolving within 24 h; mild pericardial effusion in 3/18 echo patients; no	Cardiac manifestations significantly correlated with abdominal pain, fluid accumulation, mucosal bleed, lethargy/restlessness, hepatomegaly, and features of severe dengue;

		dissociation with sinus node dysfunction 1%; ST–T changes in 11%	rhythm disturbances reverted to sinus rhythm within 24 hours.	dissociation; troponin T tested in 18 patients (all negative); echocardiography in 18 patients	myocarditis seen; 2 deaths due to ARDS; significant correlation between cardiac manifestations and all warning signs except persistent vomiting	fluid accumulation causing respiratory distress was significantly associated with cardiac involvement; all arrhythmias transient with no lasting sequelae
Vikas et al. [26]	2023	100 total; cardiovascular manifestations in 25%; sinus tachycardia 17%, sinus bradycardia 6%; complete heart block 1%; second-degree SA exit block type I (Wenckebach) 1%	Sinus tachycardia, sinus bradycardia, complete heart block, second-degree sinoatrial exit block (Wenckebach).	12-lead ECG in all patients; rhythm abnormalities described including tachycardia, bradycardia, complete heart block, SA exit block; myocarditis diagnosed via ECG + echo (LV systolic dysfunction, global hypokinesia, EF 40%)	Cardiovascular manifestations in 25%; fever in 100%; myalgia 28%; vomiting 21%; bleeding 20%; thrombocytopenia 91%; ECG changes significantly correlated with WHO severity (p=0.007); myocarditis in 1 patient; echo normal in 7/8 tested patients; Troponin I negative in all 3 tested	Arrhythmias (tachycardia, bradycardia, CHB, SA exit block) correlated significantly with dengue severity (p=0.007); CHB case required temporary pacemaker; arrhythmias more common in warning signs and severe dengue groups; myocarditis present in one patient with CHB and LV dysfunction

Table S4. Reporting for classification of dengue

		No alarm signs		With alarm sign		Severe dengue		Total dengue
**For adults**								
Study	Year	Events	Sample	Events	Sample	Events	Sample	
Arfeen [15]	2024							
Baqi [41]	2022							
Bhatt [36]	2020	0	37	8	85	5	60	182
Espinosa Brito [33]	2014							
Budhia [24]	2024							
Cabrera-Rego [35]	2021							
Dhivya [23]	2019							
Furlan-Daniel [13]	2019							
Garg [18]	2024							
Gnanamuthu [17]	2018							
Gugale [44]	2022							
Gupta [28]	2022							
Hussain [37]	2017							
Iqbal [34]	2023							

Kothendaraman [45]	2024							
Kularatne [16]	2007							
Li [19]	2016	166	1707	35	75			
Ramchandwani [20]	2024							
Kumari [12]	2024							
Shah [42]	2020							
Tabassum [40]	2023							
Thirumurugan [25]	2022							
Vikas [26]	2023	11	63	9	27	5	10	
Kollipara [46]	2025							
Ruhella [29]	2018							
Tarique [31]	2013							
Salam [30]	2022							
**For children**								
Buntubatu [38]	2019							
Chaudhary [21]	2020							
Godoy [27]	2018							
La-Fontaine-Terry [14]	2023							
La-Orkhun [43]	2011							
Nadkarni [39]	2020							
Nerella [32]	2022	13	61	29	67	20	22	
Pothapregada [22]	2016							

Severity-stratified data were reported inconsistently across included studies. Among adults, only three studies provided extractable arrhythmia data according to dengue severity, and among pediatric populations only one study reported usable stratified data. Descriptively, the proportion of arrhythmias appeared higher in patients with warning signs or severe dengue than in those without warning signs. However, because these data were sparse and unevenly reported, particularly in children, no robust age-specific severity-stratified meta-analysis was performed.

Table S5: Laboratory test confirmation

Author	Laboratory	
Arfeen [15]	YES	The study included patients of all age groups who were admitted to the hospital with a confirmed diagnosis of dengue fever, based on clinical criteria and laboratory confirmation (positive dengue NS1 antigen and/or dengue IgM antibodies)
Baqi [41]	YES	A positive dengue antigen (nonstructural protein-1, NS-1) or anti-dengue immunoglobulin M (IgM) assay (antibody-capture enzyme-<br>linked immunosorbent assay, PanBio, Brisbane, Australia) was used as the inclusion criteria [7].
Bhatt [36]	YES	The diagnosis<br>of dengue was based on NS1 ELISA/ IgM-capture enzymelinked immunosorbent assay (MAC-ELISA).
Espinosa-Brito [33]	No	with the diagnosis of DHF, according to the criteria of the WHO Expert Committee of 1975 and 1980 (That is a series of criteria but does not include serological or molecular tests)
Budhia [24]	YES	Dengue was confirmed by testing NS1 AG,<br>IgG, and IgM in the patients.
Cabrera-Rego [35]	YES	IgM confirmation
Dhivya [23]	YES	A cross-sectional study was conducted with a total of 250 patients. They were diagnosed with positive serology for dengue (NS1 or IgM) before being included in the study.

Furlan-Daniel [13]	YES	Elisa and NS1
Garg [18]	YES	Includes 385 patients aged 18 years or older with dengue confirmed by NS1 or IgM antibodies
Gnanamuthu [17]	No	Two hundred adult patients who had febrile thrombocytopenia and had been hospitalized were enrolled in the study.
Gugale [44]	YES	The 140 patients aged ≥18 years with DF confirmed with a serology-dengue non-structural protein 1 antigenpositive were included for this prospective observational study
Gupta [28]	YES	The confirmation of diagnosis of DF was made on the basis of suggestive symptoms or signs followed by positive testing by rapid diagnostic test (NS1 or IgM positive) and/or positive serology (IgM) by Enzyme Linked Immunosorbent Assay (ELISA).
Hussain [37]	No	All adult patients with Dengue fever transferred to medical ICU of Ittefaq Hospital during the epidemic of dengue fever from September 2011 to October 2011 were included.
Iqbal [34]	No	Inclusion Criteria: Patients who fulfill the following criteria were included in the study: • High grade fever between 2 to 10 days; • Thrombocytopenia and Leukopenia; • Capillary leak signs present
Kothendaraman [45]	YES	Those who presented with fever of less than five days underwent Dengue NS1 antigen testing, while those who presented after 5 days of fever were investigated for dengue IgM using ELISA.
Kularatne [16]	YES	Dengue RT-PCR-agarose gel electrophoresis (RT-PCR-AGE) was done for patients who presented within 4 days of onset of fever.
Li [19]	YES	Dengue IgM/IgG Capture enzyme-linked immunosorbent assay (ELISA; Daan Gene Co, Ltd. of Sun Yat-sen University, China), a rapid detection of NS1 (ELISA kit InBios, seattle, WA) and reverse transcription polymerase chain reaction (RT-PCR) were used to detect dengue virus infection in clinically suspected patients.
Ramchandwani [20]	YES	positive laboratory tests (NS1 antigen, IgG, or IgM).
Ruhella [29]	YES	Serologically confirmed cases of dengue fever who were admitted in S.p. medical college Bikaner Rajasthan in period between June 1, 2016 to May 30, 2017 were selected for the study

Kollipara [46]	YES	Serologically confirmed dengue patients (NS1 antigen and/or IgM positive)
Salam [30]	YES	Inclusion: high-grade fever 1–5 days, NS1/IgM/IgG positive
Shah [42]	YES	Serological diagnosis was based on Dengue IgG/IgM rapid card test
Tabassum [40]	YES	2018.Admitted patients with high grade fever and positive Dengue IgM, NS1 AG or RT-PCR were included.
Tarique [31]	YES	positive laboratory tests (NS1 antigen, IgG, or IgM).
Thirumurugan [25]	YES	ELISA para dengue IgM
Vikas [26]	YES	Confirmed dengue fever cases with a positive NS1 Ag and/or IgM result that meet the WHO criteria for dengue fever.
Buntubatu [38]	YES	NS1 AND IGM
Chaudhary [21]	YES	Dengue confirmed by NS1 antigen and/or IgM antibodies
Godoy [27]	YES	NS1
La-Fontaine-Terry [14]	YES	immunoglobulin M (IgM)
La-Orkhun [43]	YES	ELISA y PCR
Nadkarni [39]	YES	NS1 +, IgM por ELISA +
Nerella [32]	YES	NS1 AND IGM

Pothapregada [22]	YES	NS1 AND IGM

Table S6. Summary of Risk of Bias Assessment (JBI Checklist)

Study	Item 1	Item 2	Item 3	Item 4	Item 5	Item 6	Item 7	Item 8	Item 9	Overall appraisal	
Arfeen et al. 2024 [15]	Yes	Yes	Unclerr*	Yes	Yes	Yes	No †	Yes	Yes	Moderate RoB	*Does not show calculus<br>†Only ECG testing if symptomatic
Baqi et al. 2022 [41]	Yes	Yes	Yes	Yes	Yes	Yes	No*	Yes	Yes	Moderate RoB	*Only ECG testing if symptomatic
Bhatt et al. 2020 [36]	Yes	Yes	Yes	Yes	Yes	Yes	Yes	Yes	Yes	Low RoB	Not applicable
Budhia et al. 2024 [24]	Yes	Yes	No*	Yes	Yes	Yes	Unclea†	Unclea‡	Yes	High RoB	*Small sample size and no Calculus<br>†Outcome measurement lacked uniform application<br>‡Prevalence paper that only reports percentages without IC95
Buntubatu et al. 2019 [38]	Yes	Yes	Yes	Yes	No*	Yes	Yes	No †	Yes	Low RoB	*Small part of the sample could not be tested with an EKG<br>† Prevalence estimates reported without confidence intervals
Cabrera-Rego et al. 2021 [35]	Yes	Unclear *	Yes	Yes	No †	Yes	Yes	Yes	No ¶	High RoB	*Large proportion of eligible patients was excluded, cannot verify true consecutive sampling<br>†Incomplete coverage of eligible patients<br>Less than 50% response rate
Chaudhary et al. 2020 [21]	Yes	Unclear *	No †	Yes	Yes	Yes	Unclear‡	Yes	Unclear ¶	Moderate RoB	*Although prospective, no mention of consecutive sampling<br>† Small sample and no calculus<br>‡ ECG not standardized; no diagnostic criteria for Arrythmia<br>¶ Response rate and screening numbers not reported

Dhivya et al. 2019 [23]	Yes	No*	Yes	Yes	No †	Yes	Unclear‡	Unclear¶	No Δ	High RoB	*Purposive sampling was used<br>†No specification of total patients screened, cannot confirm complete coverage<br>‡Cannot verify if ECG was performed at basal or only if symptomatic<br>¶As a pure descriptive prevalence paper it only only reports percentages without IC95<br>Δ Cannot calculate response rate
Espinosa-Brito 2014 [33]	Yes	Yes	Yes	Yes	Yes	Yes	No*	Yes	Yes	Moderate RoB	*Part of the sample did not undergo ECG
Furlan-Daniel et al. 2019 [13]	Yes	Yes	Unclear*	Yes	Yes	Yes	Yes	Yes	Yes	Low RoB	* Moderate sample size and no calculus
Garg et al. 2024 [18]	Yes	Yes	Yes	Yes	Yes	Yes	Yes	Yes	Yes	Low RoB	Not applicable
Gnanamuthu et al. 2018 [17]	No*	Unclear †	Yes	Unclear‡	Yes	No ¶	No Δ	Yes	Unclear±	High RoB	* Sample frame inappropriate for dengue- specific populations<br>† Sampling procedure not described; consecutive recruitment cannot be verified<br>‡ Setting description insufficient; population characteristics minimally reported<br>¶ Impossible to determine if dengue was laboratory confirmed<br>Δ No standardized definition of ECG changes<br>± Lack of information regarding response rate
Godoy et al. 2018 [27]	Yes	Yes	Yes	Yes	Yes	Yes	Unclear *	Yes	Unclear†	Moderate RoB	*Although ECG was performed, the study does not specify whether bradycardia was identified clinically or

											defined by ECG parameters. No diagnostic criteria for arrythmia<br>† Response rate and screening numbers not<br>reported
Gugale et al. 2022 [44]	Yes	Yes	Yes	Yes	Yes	Yes	Yes	Yes	Yes	Low RoB	Not applicable
Gupta et al. 2022 [28]	Yes	Unclear*	Unclear†	Yes	Yes	Yes	Yes	Yes	Yes	Moderate RoB	* Consecutive sampling not stated; enrolment process unclear.<br>† Moderate sample size and no calculus
Hussain et al. 2017 [37]	No*	No †	No ‡	Unclear¶	NoΔ	Unclea±	No•	Yes	No°	High RoB	*Sample limited to only ICU shock patients<br>† Retrospective selection without Reported consecutive recruitment<br>‡ No sample size calculation, small retrospective series<br>¶ Setting described superficially Δ Unclear whether all patients with suspected myocarditis were included<br>± ECG lacked standard definition, retrospective records withour consistents protocols<br>• Less than 50% response rate<br>° Eligibility numbers and case identification pathway not reported
Iqbal et al. 2023 [34]	No	No †	No ‡	Unclear ¶	NoΔ	No ±	No •	Yes	Unclear°	High RoB	*Sample limited to patients with dengue hemorrhagic fever<br>† Retrospective selection; no evidence of consecutive recruitment<br>‡Small sample size and no calculation<br>¶ Setting and population poorly described<br>Δ Coverage incomplete; unclear whether all eligible DHF patients

											included<br>± Diagnosis based on clinical leak criteria; lab confirmation not explicitly reported for all cases<br>• ECG/Echo performed only in selected case, no standarized definition for arryhtmia<br>° Response rate not reported
Kothendaraman et al. 2024 [45]	Yes	Unclear*	No †	Yes	Yes	Yes	Yes	Yes	Unclear‡	Low RoB	*Although prospective, no mention of consecutive sampling<br>† Small sample size and no calculation<br>‡ Response rate and screening numbers not<br>reported
Kularatne et al. 2007 [16]	No *	Unclear†	No ‡	Yes	Yes	Yes	Yes	Yes	Unclear¶	Moderate RoB	*Sample limited to patients with dengue fever (DF) and outbreak setting<br>† Consecutive recruitment not reported; selection pathway<br>Unclear ‡ Small sample size and no calculation.<br>¶ Eligibility and exclusion details incomplete
La-Fontaine-Terry et al. 2023 [14]	Yes	Yes	Yes	Yes	Yes	Yes	Yes	Yes	Unclear†	Low RoB	*Response rate and screening numbers not reported
La-Orkhun et al. 2011 [43]	Yes	Unclear *	No †	Yes	Yes	Yes	Yes	Yes	Unclear	Moderate RoB	* Consecutive sampling not stated; enrolment process unclear.<br>† Small sample and no calculus<br>‡ Response rate and screening numbers not reported
Li et al. 2016 [19]	No*	Yes	Yes	Yes	No †	Yes	Yes	No ‡	No ¶	Moderate RoB	*Outbreak setting<br>† Not all patients with abnormal ECG were included<br>‡ Prevalence estimates reported without confidence intervals

											¶ Less than 80% of abnormal ECG were analyzed; missing cases not explained
Nadkarni et al. 2020 [39]	Yes	Unclear *	No †	Yes	Yes	Yes	No ‡	Yes	Yes	High RoB	* Consecutive sampling not stated; enrolment process unclear<br>† Small sample and no calculus<br>‡ Only ECG testing if symptomatic
Nerella et al. 2022 [32]	Yes	Unclear *	Unclear†	Yes	Yes	Yes	Yes	Yes	Yes	Moderate RoB	*Although prospective, no mention of consecutive sampling<br>† Moderate sample size, no calculus
Pothapregada et al. 2016 [22]	Yes	Yes	Yes	Unclear*	Yes	Yes	Unclear†	Yes	Unclear	Moderate RoB	*Setting and population poorly described<br>† Study does not specify whether bradycardia was identified clinically or defined by ECG parameters. No diagnostic criteria for arrythmia<br>‡ Response rate and screening numbers not reported
Ramchandwani et al. 2024 [20]	Yes	Unclear*	No †	Yes	Yes	Yes	Unclear‡	Yes	Unclear¶	Moderate RoB	* Consecutive recruitment not reported; selection pathway<br>Unclear † Small sample size and no calculus<br>‡ ECG not standardized; no diagnostic criteria<br>¶ No reporting of screened population
Kumari et al. 2024 [12]	Yes	Unclear *	No †	Yes	Yes	Yes	Unclear‡	No ¶	UnclearΔ	Moderate RoB	* Consecutive sampling not reported; unclear recruitment pathway<br>† Small sample size and no calculation.<br>‡ ECG not standardized; no diagnostic criteria<br>¶ Prevalence estimates reported without confidence intervals<br>Δ Response rate and screening numbers not reported
Shah et al. 2021 [42]	Yes	Unclear *	Yes	Yes	Yes	Yes	Unclear†	Yes	Yes	Moderate RoB	* Consecutive sampling not stated; Enrolment process unclear.

											† No diagnostic criteria for arryhtmia mentioned
Tabassum et al. 2023 [40]	Yes	Yes	No*	Yes	Yes	Yes	Yes	No	Yes	Low RoB	*Small sample size, authors mention their calculated sample was much larger<br>† Prevalence estimates reported without confidence intervals
Thirumurugan et al. 2022 [25]	Yes	Yes	Yes	Yes	Yes	Yes	Unclear *	No †	Unclear‡	Moderate RoB	*ECG not standardized; no diagnostic criteria for arrythmia<br>† Prevalence estimates reported without confidence intervals<br>‡ Response rate and screening numbers not reported
Vikas et al. 2023 [26]	Yes	Unckear *	Yes	Yes	Yes	Yes	Unclear †	Yes	Unclear ‡	Moderate RoB	* Consecutive sampling not stated; enrollment process unclear.<br>† ECG not standardized; no diagnostic criteria for arrythmia<br>‡ Response rate and screening numbers not reported

Overall, the methodological quality of the 35 included studies was moderate, with substantial variability across JBI domains. Eight studies were judged to have low risk of bias, eighteen moderate risk of bias, and seven high risk of bias. Most studies adequately described their setting and diagnostic criteria for dengue (Items 1, 4, and 6), but important limitations were repeatedly observed in sampling methods, standardization of cardiac assessments, and statistical precision. Nineteen studies did not report consecutive sampling or an adequate recruitment pathway, resulting in frequent "Unclear" ratings for Item 2. Eighteen studies had either a small sample size or no calculation for their reported sample (Item 3: predominantly "No").

The most recurrent methodological concern was non-standardized measurement of cardiac outcomes (Item 7). Twenty studies did not specify the context in which arrhythmias were evaluated, such as whether a trained reader assessed the tracings, or whether diagnostic thresholds for bradycardia, tachycardia, atrioventricular blocks or ST abnormalities were defined. Others performed ECG only in selected subgroups (e.g., symptomatic patients or those with warning signs). For studies reporting the prevalence of cardiac abnormalities, none provided 95% confidence intervals, which led to consistent "No" ratings for Item 8 in accordance with JBI criteria for statistical adequacy. Reporting of response rates and screening denominators was also frequently incomplete (Item 9: often "Unclear"), particularly in retrospective studies or those conducted during outbreak conditions.

Table S7. GRADE Certainty of evidence

№ of studies	Certainty assessment						impact	Certainty	Importance
	Study design	Risk of bias	Inconsistency	Indirectness	Imprecision	Other considerations			
Prevalence of any arryhtmia (assessed with: 12-lead ECG)									
35	non-randomised studies	serious[a]	serious[b]	not serious	not serious	none	24.48% (95% CI 17.54–33.07)	⊕⊕◯◯ Low[a,b]	CRITICAL
Bradycardia (assessed with: 12-lead ECG)									
26	non-randomised studies	serious[a]	serious[b]	not serious	not serious	none	11.84% (CI 95% 8.63 - 16.03)	⊕⊕◯◯ Low[a,b]	IMPORTANT
Tachycardia (assessed with: 12-lead ECG)									
19	non-randomised studies	serious[a]	serious[b]	not serious	serious[c]	none	10.63% (CI 95% 6.41 - 17.13)	⊕◯◯◯ Very low[a,b,c]	IMPORTANT
Atrioventricular block (assessed with: 12-lead ECG)									
11	non-randomised studies	serious[a]	not serious	not serious	serious[d]	none	1.33% (CI 95% 0.81 - 2.18)	⊕⊕◯◯ Low[a,d]	CRITICAL
Other arrythmias (assessed with: 12-lead ECG)									
11	non-randomised studies	serious[a]	not serious	not serious	not serious	none	2.80% (CI 95% 1.77 - 4.40)	⊕⊕⊕◯ Moderate[a]	NOT IMPORTANT

Supplementary Figure S1A and Figure S1B: Funnel Plot

Figure S1

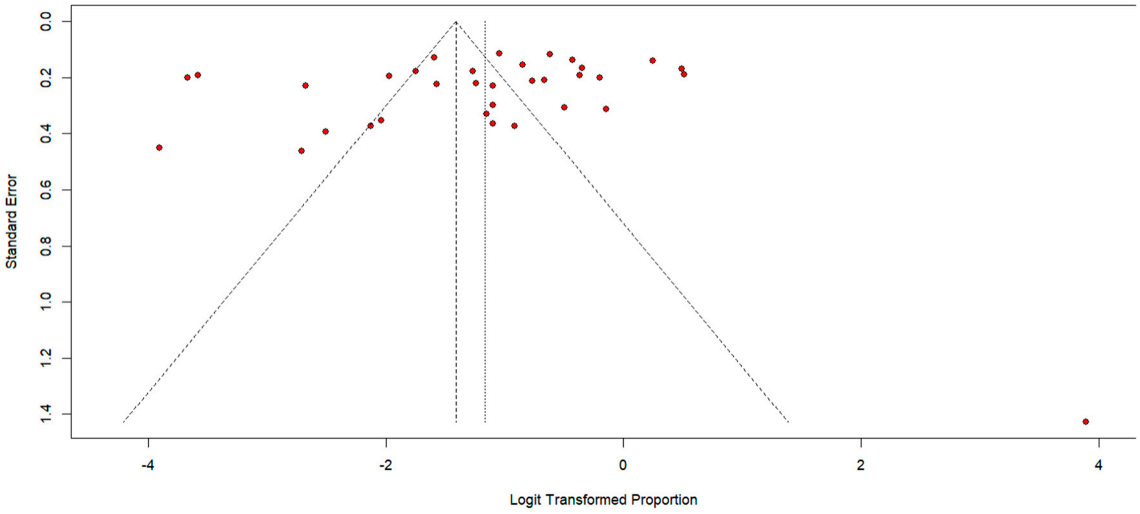

(**A**)

```
Eggers' test of the intercept
=============================

 intercept     95% CI      t          p
   -3.047 -7.6 - 1.5 -1.312 0.1984856

Eggers' test does not indicate the presence of funnel plot asymmetry.
>
```

(**B**)

Figure S1. (A) Funnel plot for the pooled proportion of any cardiac arrhythmia in dengue. Funnel plot showing the logit-transformed study-specific proportions of any cardiac arrhythmia among patients with dengue plotted against their standard errors. The vertical reference line represents the pooled effect estimate, and the dashed triangular limits represent the expected 95% confidence region around the pooled estimate. Visual inspection showed no clear evidence of substantial funnel plot asymmetry. **(B)** Egger's regression test for funnel plot asymmetry. Egger's test of the intercept for the logit-transformed proportion of any cardiac arrhythmia in dengue showed no statistically significant evidence of funnel plot asymmetry (intercept = −3.047; 95% CI −7.6 to 1.5; t = −1.312; p = 0.198). This suggests that small-study effects were unlikely to substantially explain the pooled estimate.

Sensitivity Analysis and Influence analisis

1. Baujat plots

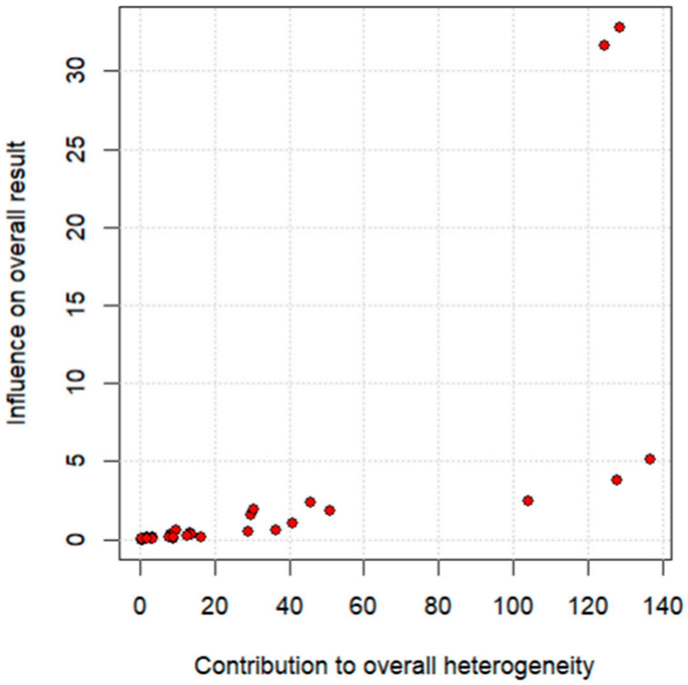

Figure S2. Baujat plot for the pooled proportion of any cardiac arrhythmia in dengue. Baujat plot showing each study's contribution to the overall between-study heterogeneity on the x-axis and its influence on the pooled effect estimate on the y-axis. Studies located in the upper-right area were considered potentially influential because they contributed substantially to heterogeneity and had a greater impact on the overall pooled estimate.

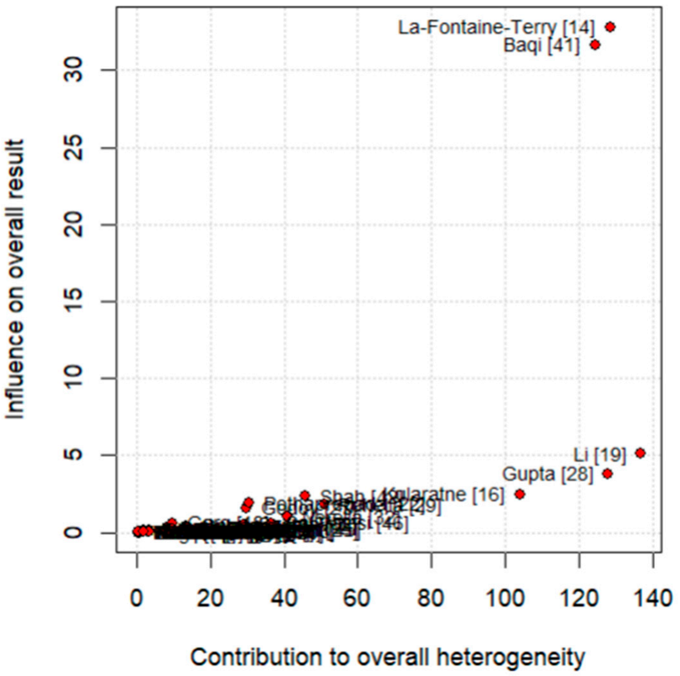

Figure S3. Labeled Baujat plot for the pooled proportion of any cardiac arrhythmia in dengue. Labeled Baujat plot showing each study's contribution to the overall between-study heterogeneity

on the x-axis and its influence on the pooled effect estimate on the y-axis. The plot identifies potentially influential studies, particularly La-Fontaine-Terry et al. [14] and Baqi et al. [41], which showed the greatest influence on the overall pooled estimate, while Li et al. [19], Gupta et al. [28], and Kularatne et al. [16] contributed substantially to heterogeneity.

1. Without Baqi et al

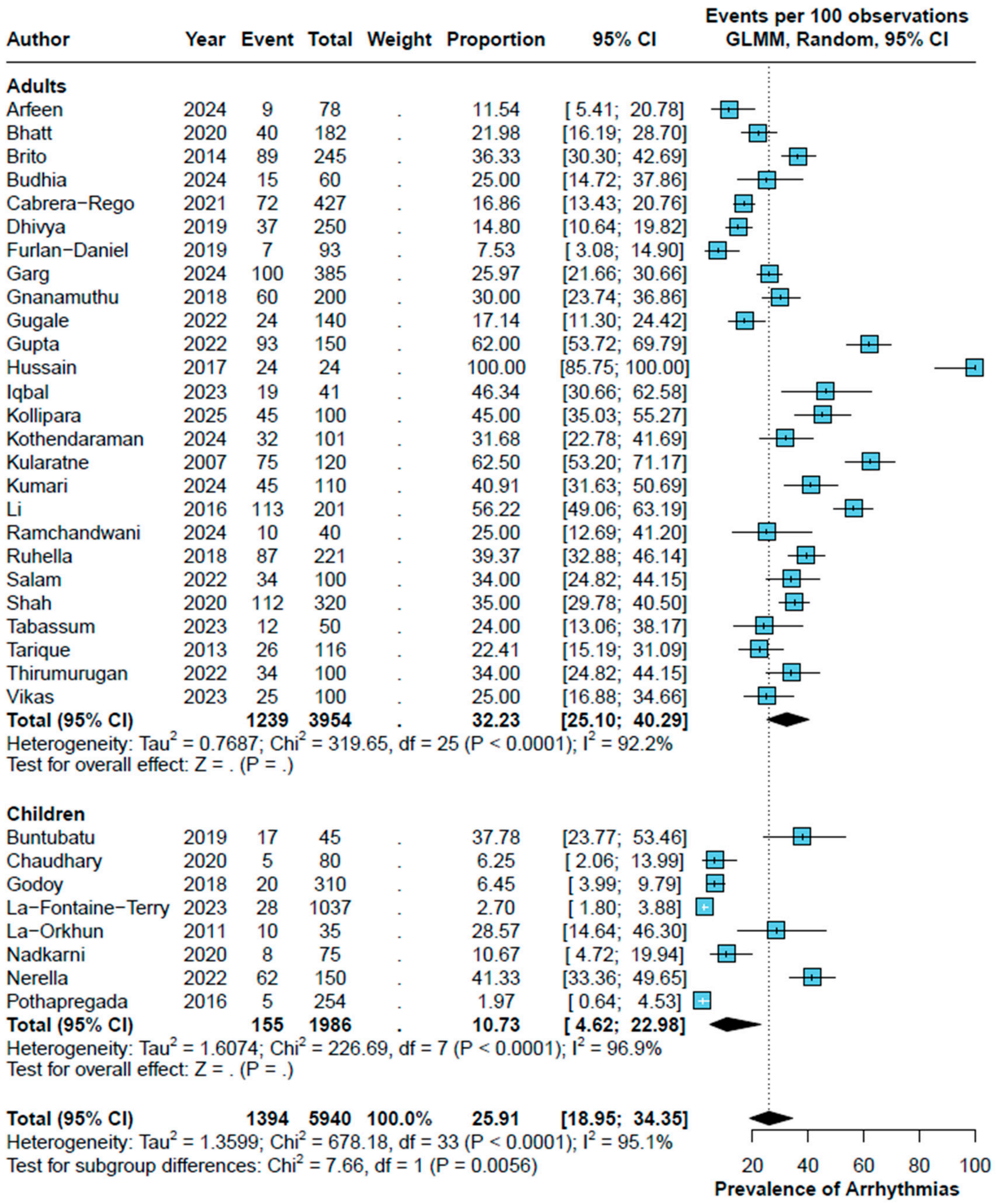

Figure S4. Sensitivity analysis excluding Baqi et al. [41]. Forest plot showing the pooled proportion of any cardiac arrhythmia in patients with dengue after excluding Baqi et al. [41], stratified by age group (adults vs. children). The analysis was performed using a random-effects generalized linear mixed model (GLMM) with logit transformation. Estimates are expressed as events per 100 patients with corresponding 95% confidence intervals. The studies included in this sensitivity analysis correspond to references [12–40,42–46].

Figure S4

2. Without La-Fontaine-Terry

Author	Year	Event	Total	Weight	Proportion	95% CI
**Adults**						
Arfeen	2024	9	78	.	11.54	[5.41; 20.78]
Baqi	2022	25	1008	.	2.48	[1.61; 3.64]
Bhatt	2020	40	182	.	21.98	[16.19; 28.70]
Brito	2014	89	245	.	36.33	[30.30; 42.69]
Budhia	2024	15	60	.	25.00	[14.72; 37.86]
Cabrera−Rego	2021	72	427	.	16.86	[13.43; 20.76]
Dhivya	2019	37	250	.	14.80	[10.64; 19.82]
Furlan−Daniel	2019	7	93	.	7.53	[3.08; 14.90]
Garg	2024	100	385	.	25.97	[21.66; 30.66]
Gnanamuthu	2018	60	200	.	30.00	[23.74; 36.86]
Gugale	2022	24	140	.	17.14	[11.30; 24.42]
Gupta	2022	93	150	.	62.00	[53.72; 69.79]
Hussain	2017	24	24	.	100.00	[85.75; 100.00]
Iqbal	2023	19	41	.	46.34	[30.66; 62.58]
Kollipara	2025	45	100	.	45.00	[35.03; 55.27]
Kothendaraman	2024	32	101	.	31.68	[22.78; 41.69]
Kularatne	2007	75	120	.	62.50	[53.20; 71.17]
Kumari	2024	45	110	.	40.91	[31.63; 50.69]
Li	2016	113	201	.	56.22	[49.06; 63.19]
Ramchandwani	2024	10	40	.	25.00	[12.69; 41.20]
Ruhella	2018	87	221	.	39.37	[32.88; 46.14]
Salam	2022	34	100	.	34.00	[24.82; 44.15]
Shah	2020	112	320	.	35.00	[29.78; 40.50]
Tabassum	2023	12	50	.	24.00	[13.06; 38.17]
Tarique	2013	26	116	.	22.41	[15.19; 31.09]
Thirumurugan	2022	34	100	.	34.00	[24.82; 44.15]
Vikas	2023	25	100	.	25.00	[16.88; 34.66]
**Total (95% CI)**		**1264**	**4962**	**.**	**30.00**	**[22.12; 39.26]**
Heterogeneity: $Tau^2 = 1.1237$; $Chi^2 = 523.16$, df = 26 (P < 0.0001); $I^2 = 95\%$						
Test for overall effect: Z = . (P = .)						
**Children**						
Buntubatu	2019	17	45	.	37.78	[23.77; 53.46]
Chaudhary	2020	5	80	.	6.25	[2.06; 13.99]
Godoy	2018	20	310	.	6.45	[3.99; 9.79]
La−Orkhun	2011	10	35	.	28.57	[14.64; 46.30]
Nadkarni	2020	8	75	.	10.67	[4.72; 19.94]
Nerella	2022	62	150	.	41.33	[33.36; 49.65]
Pothapregada	2016	5	254	.	1.97	[0.64; 4.53]
**Total (95% CI)**		**127**	**949**	**.**	**13.09**	**[5.61; 27.65]**
Heterogeneity: $Tau^2 = 1.4567$; $Chi^2 = 123.18$, df = 6 (P < 0.0001); $I^2 = 95.1\%$						
Test for overall effect: Z = . (P = .)						
**Total (95% CI)**		**1391**	**5911**	**100.0%**	**25.87**	**[18.88; 34.34]**
Heterogeneity: $Tau^2 = 1.3753$; $Chi^2 = 668.98$, df = 33 (P < 0.0001); $I^2 = 95.1\%$						
Test for subgroup differences: $Chi^2 = 4.05$, df = 1 (P = 0.0441)						

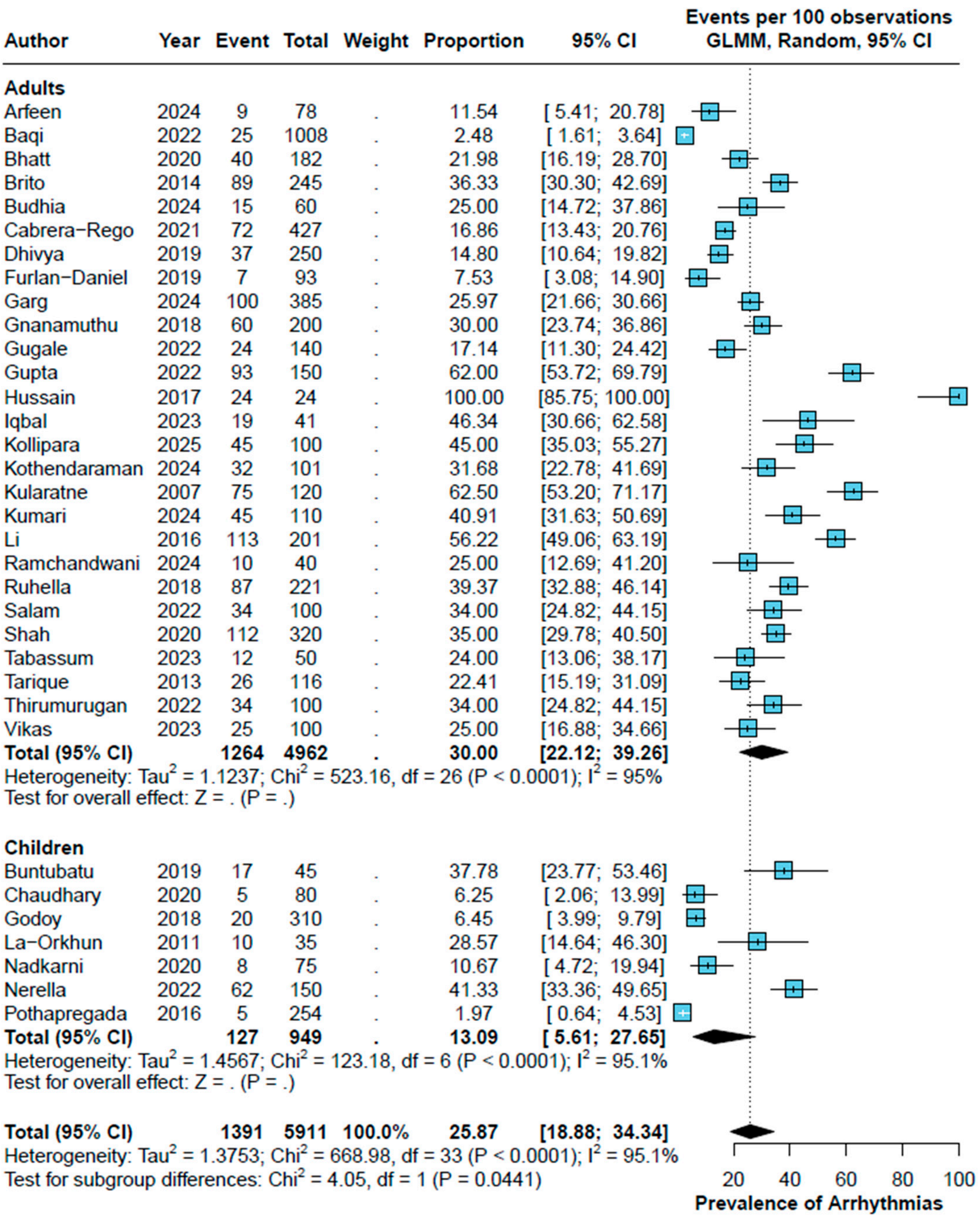

Figure S5. Sensitivity analysis excluding La-Fontaine-Terry et al. [14]. Forest plot showing the pooled proportion of any cardiac arrhythmia in patients with dengue after excluding La-Fontaine-Terry et al. [14], stratified by age group (adults vs. children). The analysis was performed using a random-effects generalized linear mixed model (GLMM) with logit transformation. Estimates are expressed as events per 100 patients with corresponding 95% confidence intervals. The studies included in this sensitivity analysis correspond to references [12,13,15–46].

3. Without both

Author	Year	Event	Total	Weight	Proportion	95% CI
**Adults**						
Arfeen	2024	9	78	.	11.54	[5.41; 20.78]
Bhatt	2020	40	182	.	21.98	[16.19; 28.70]
Brito	2014	89	245	.	36.33	[30.30; 42.69]
Budhia	2024	15	60	.	25.00	[14.72; 37.86]
Cabrera-Rego	2021	72	427	.	16.86	[13.43; 20.76]
Dhivya	2019	37	250	.	14.80	[10.64; 19.82]
Furlan-Daniel	2019	7	93	.	7.53	[3.08; 14.90]
Garg	2024	100	385	.	25.97	[21.66; 30.66]
Gnanamuthu	2018	60	200	.	30.00	[23.74; 36.86]
Gugale	2022	24	140	.	17.14	[11.30; 24.42]
Gupta	2022	93	150	.	62.00	[53.72; 69.79]
Hussain	2017	24	24	.	100.00	[85.75; 100.00]
Iqbal	2023	19	41	.	46.34	[30.66; 62.58]
Kollipara	2025	45	100	.	45.00	[35.03; 55.27]
Kothendaraman	2024	32	101	.	31.68	[22.78; 41.69]
Kularatne	2007	75	120	.	62.50	[53.20; 71.17]
Kumari	2024	45	110	.	40.91	[31.63; 50.69]
Li	2016	113	201	.	56.22	[49.06; 63.19]
Ramchandwani	2024	10	40	.	25.00	[12.69; 41.20]
Ruhella	2018	87	221	.	39.37	[32.88; 46.14]
Salam	2022	34	100	.	34.00	[24.82; 44.15]
Shah	2020	112	320	.	35.00	[29.78; 40.50]
Tabassum	2023	12	50	.	24.00	[13.06; 38.17]
Tarique	2013	26	116	.	22.41	[15.19; 31.09]
Thirumurugan	2022	34	100	.	34.00	[24.82; 44.15]
Vikas	2023	25	100	.	25.00	[16.88; 34.66]
**Total (95% CI)**		**1239**	**3954**	**.**	**32.23**	**[25.10; 40.29]**
Heterogeneity: $Tau^2 = 0.7687$; $Chi^2 = 319.65$, df = 25 ($P < 0.0001$); $I^2 = 92.2\%$						
Test for overall effect: Z = . (P = .)						
**Children**						
Buntubatu	2019	17	45	.	37.78	[23.77; 53.46]
Chaudhary	2020	5	80	.	6.25	[2.06; 13.99]
Godoy	2018	20	310	.	6.45	[3.99; 9.79]
La-Orkhun	2011	10	35	.	28.57	[14.64; 46.30]
Nadkarni	2020	8	75	.	10.67	[4.72; 19.94]
Nerella	2022	62	150	.	41.33	[33.36; 49.65]
Pothapregada	2016	5	254	.	1.97	[0.64; 4.53]
**Total (95% CI)**		**127**	**949**	**.**	**13.09**	**[5.61; 27.65]**
Heterogeneity: $Tau^2 = 1.4567$; $Chi^2 = 123.18$, df = 6 ($P < 0.0001$); $I^2 = 95.1\%$						
Test for overall effect: Z = . (P = .)						
**Total (95% CI)**		**1366**	**4903**	**100.0%**	**27.45**	**[20.58; 35.59]**
Heterogeneity: $Tau^2 = 1.1583$; $Chi^2 = 473.79$, df = 32 ($P < 0.0001$); $I^2 = 93.2\%$						
Test for subgroup differences: $Chi^2 = 5.13$, df = 1 ($P = 0.0234$)						

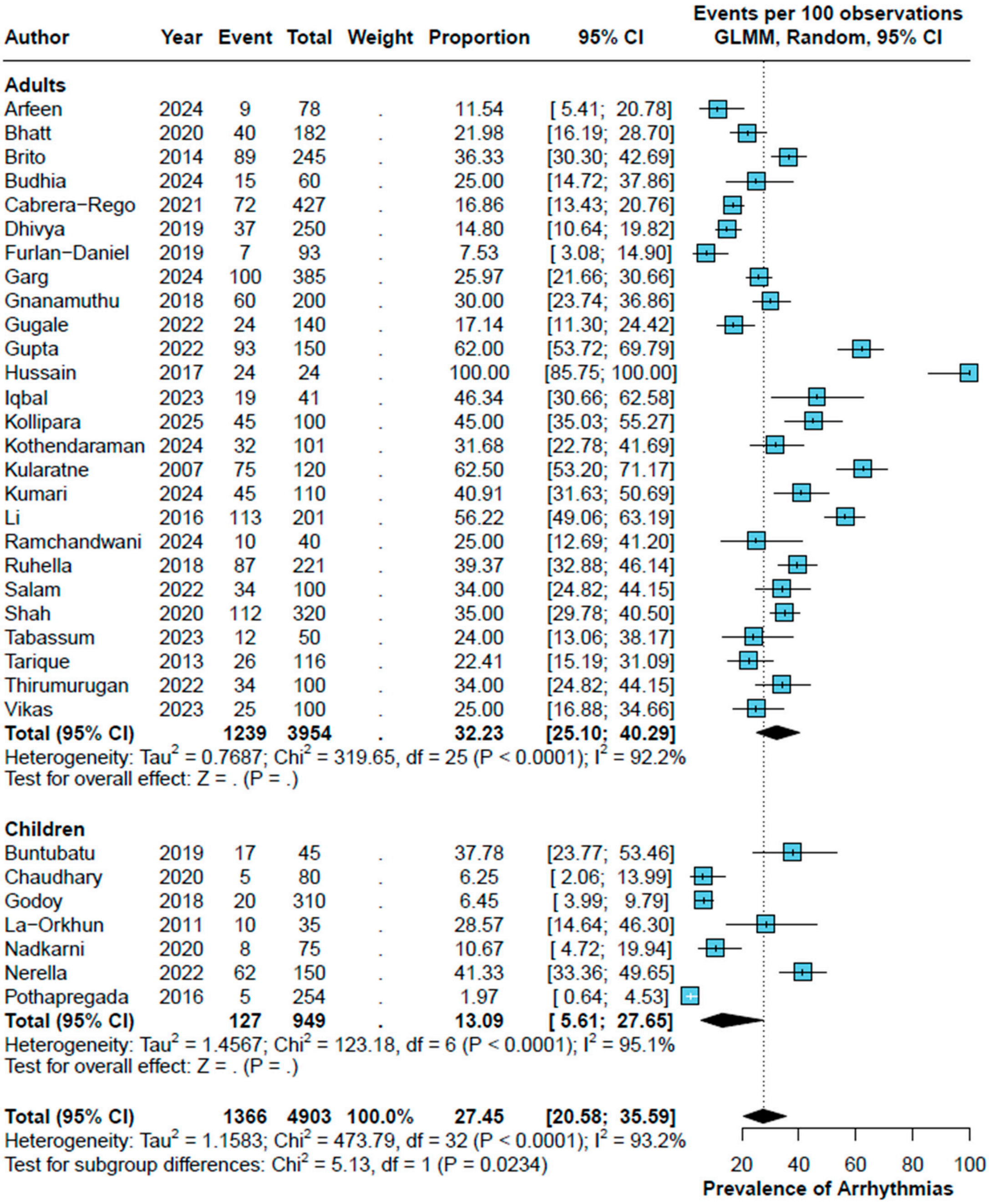

Figure S6. Sensitivity analysis excluding Baqi et al. [41] and La-Fontaine-Terry et al. [14]. Forest plot showing the pooled proportion of any cardiac arrhythmia in patients with dengue after excluding Baqi et al. [41] and La-Fontaine-Terry et al. [14], stratified by age group (adults vs. children). The analysis was performed using a random-effects generalized linear mixed model (GLMM) with logit transformation. Estimates are expressed as events per 100 patients with corresponding 95% confidence intervals. The studies included in this sensitivity analysis correspond to references [12,13,15–40,42–46].

Analysis

The influential-study sensitivity analyses showed that excluding Baqi et al., La-Fontaine-Terry et al., or both studies together did not materially change the overall interpretation of the meta-analysis. Compared with the primary pooled estimate of 24.48% (95% CI 17.54–33.07), the pooled proportion was 25.91% (95% CI 18.95–34.35) after excluding Baqi et al., 25.87% (95% CI 18.88–34.34) after excluding La-Fontaine-Terry et al., and 27.45% (95% CI 20.58–35.59) after excluding both studies. Although the pooled estimate shifted upward, the confidence intervals overlapped substantially with those of the primary analysis, and the clinical interpretation

remained unchanged. This upward shift was expected because both excluded studies reported relatively low arrhythmia proportions and had large sample sizes, thereby pulling the overall estimate downward in the primary model. Importantly, cardiac arrhythmias continued to be observed in approximately one quarter of patients with dengue, and the subgroup pattern of a higher pooled proportion in adults than in children persisted across all sensitivity analyses. Heterogeneity decreased slightly after these exclusions but remained high (I^2 = 95.1%, 95.1%, and 93.2%, respectively), indicating that these two studies contributed to heterogeneity and statistical influence but were not the sole drivers of the between-study variability.

2. Comparison between GLMM and Freeman-Tukey method

Generalized linear mixed model (GLMM)

Author	Year	Event	Total	Weight	Proportion	95% CI
**Adults**						
Arfeen	2024	9	78	.	11.54	[5.41; 20.78]
Baqi	2022	25	1008	.	2.48	[1.61; 3.64]
Bhatt	2020	40	182	.	21.98	[16.19; 28.70]
Brito	2014	89	245	.	36.33	[30.30; 42.69]
Budhia	2024	15	60	.	25.00	[14.72; 37.86]
Cabrera-Rego	2021	72	427	.	16.86	[13.43; 20.76]
Dhivya	2019	37	250	.	14.80	[10.64; 19.82]
Furlan-Daniel	2019	7	93	.	7.53	[3.08; 14.90]
Garg	2024	100	385	.	25.97	[21.66; 30.66]
Gnanamuthu	2018	60	200	.	30.00	[23.74; 36.86]
Gugale	2022	24	140	.	17.14	[11.30; 24.42]
Gupta	2022	93	150	.	62.00	[53.72; 69.79]
Hussain	2017	24	24	.	100.00	[85.75; 100.00]
Iqbal	2023	19	41	.	46.34	[30.66; 62.58]
Kollipara	2025	45	100	.	45.00	[35.03; 55.27]
Kothendaraman	2024	32	101	.	31.68	[22.78; 41.69]
Kularatne	2007	75	120	.	62.50	[53.20; 71.17]
Kumari	2024	45	110	.	40.91	[31.63; 50.69]
Li	2016	113	201	.	56.22	[49.06; 63.19]
Ramchandwani	2024	10	40	.	25.00	[12.69; 41.20]
Ruhella	2018	87	221	.	39.37	[32.88; 46.14]
Salam	2022	34	100	.	34.00	[24.82; 44.15]
Shah	2020	112	320	.	35.00	[29.78; 40.50]
Tabassum	2023	12	50	.	24.00	[13.06; 38.17]
Tarique	2013	26	116	.	22.41	[15.19; 31.09]
Thirumurugan	2022	34	100	.	34.00	[24.82; 44.15]
Vikas	2023	25	100	.	25.00	[16.88; 34.66]
**Total (95% CI)**		**1264**	**4962**	**.**	**30.00**	**[22.12; 39.26]**
Heterogeneity: Tau^2 = 1.1237; Chi^2 = 523.16, df = 26 (P < 0.0001); I^2 = 95%						
Test for overall effect: Z = . (P = .)						
**Children**						
Buntubatu	2019	17	45	.	37.78	[23.77; 53.46]
Chaudhary	2020	5	80	.	6.25	[2.06; 13.99]
Godoy	2018	20	310	.	6.45	[3.99; 9.79]
La-Fontaine-Terry	2023	28	1037	.	2.70	[1.80; 3.88]
La-Orkhun	2011	10	35	.	28.57	[14.64; 46.30]
Nadkarni	2020	8	75	.	10.67	[4.72; 19.94]
Nerella	2022	62	150	.	41.33	[33.36; 49.65]
Pothapregada	2016	5	254	.	1.97	[0.64; 4.53]
**Total (95% CI)**		**155**	**1986**	**.**	**10.73**	**[4.62; 22.98]**
Heterogeneity: Tau^2 = 1.6074; Chi^2 = 226.69, df = 7 (P < 0.0001); I^2 = 96.9%						
Test for overall effect: Z = . (P = .)						
**Total (95% CI)**		**1419**	**6948**	**100.0%**	**24.48**	**[17.54; 33.07]**
Heterogeneity: Tau^2 = 1.5426; Chi^2 = 861.99, df = 34 (P < 0.0001); I^2 = 96.1%						
Test for subgroup differences: Chi^2 = 6.24, df = 1 (P = 0.0125)						

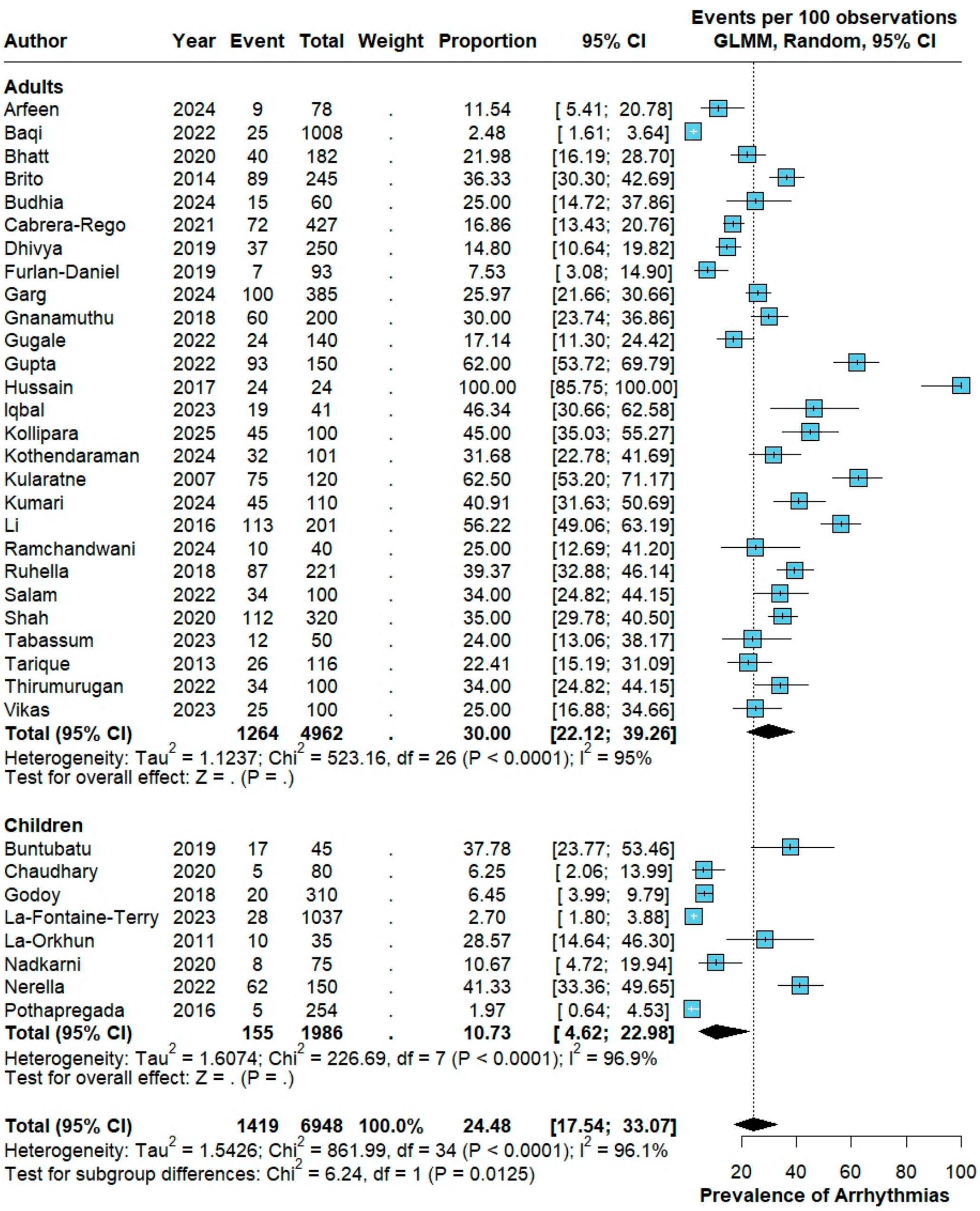

Figure S7. Pooled proportion of any cardiac arrhythmia in dengue using the generalized linear mixed model. Forest plot showing the pooled proportion of any cardiac arrhythmia among

patients with dengue, stratified by age group (adults vs. children), using a random-effects generalized linear mixed model (GLMM) with logit transformation. Estimates are expressed as events per 100 patients with corresponding 95% confidence intervals. This analysis was used as the primary model and compared with the Freeman–Tukey double-arcsine method shown in Supplementary Figure S8. The studies included in this analysis correspond to references [12–46].

Fi
Freeman Tukey

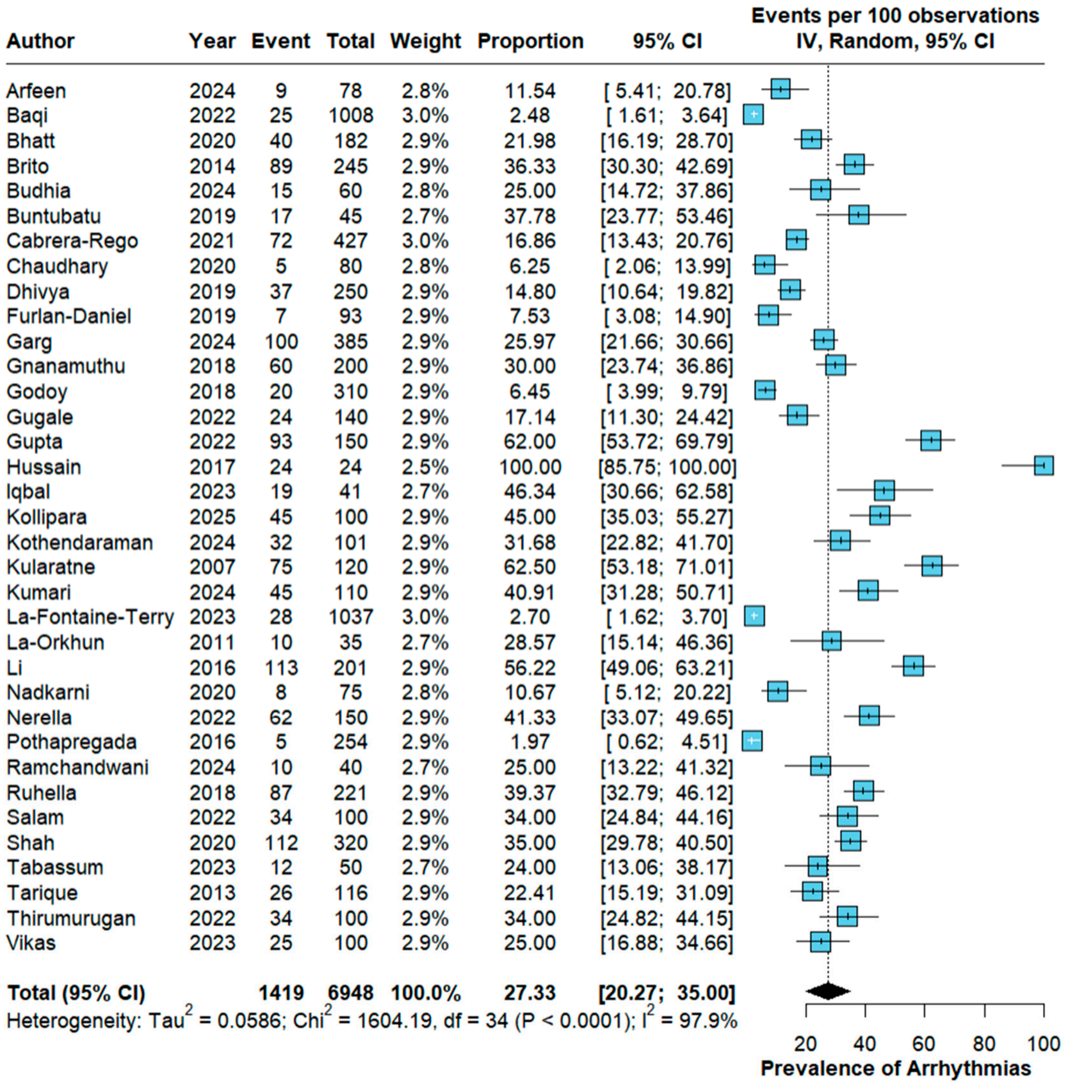

Figure S8. Pooled proportion of any cardiac arrhythmia in dengue using the Freeman–Tukey double-arcsine method. Forest plot showing the pooled proportion of any cardiac arrhythmia among patients with dengue, estimated using the Freeman–Tukey double-arcsine transformation with an inverse-variance random-effects model. Estimates are expressed as events per 100 patients with corresponding 95% confidence intervals. This analysis was performed as a sensitivity analysis and compared with the generalized linear mixed model (GLMM) shown in Supplementary Figure S7. The studies included in this analysis correspond to references [12–46].

Analysis
The sensitivity analysis using the Freeman–Tukey double-arcsine transformation yielded results broadly consistent with the primary GLMM model. The Freeman–Tukey method produced a slightly higher pooled estimate (27.33%, 95% CI 20.27–35.00) than the primary GLMM analysis (24.48%, 95% CI 17.54–33.07). However, the absolute difference was modest, and the confidence intervals overlapped substantially, indicating that the overall interpretation remained unchanged. In both analyses, cardiac arrhythmias were observed in approximately one quarter of patients with

dengue. Importantly, heterogeneity remained very high with both methods ($I^2 = 96.1\%$ for GLMM and $I^2 = 97.9\%$ for Freeman–Tukey), suggesting that the choice of transformation did not explain the substantial between-study variability.

3. Subgroup Analysis

1. For region

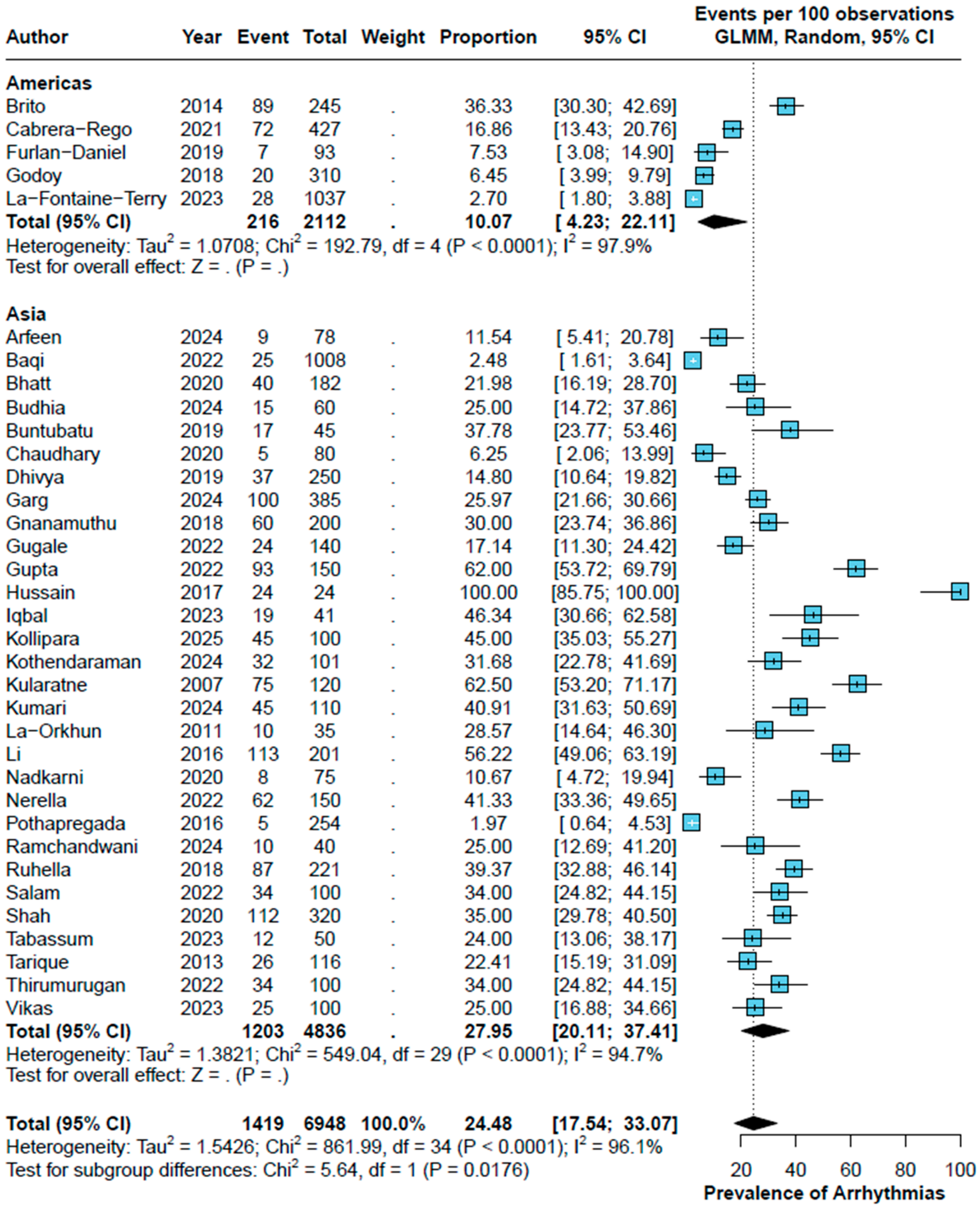

Figure S9. Subgroup analysis of the pooled proportion of any cardiac arrhythmia in dengue by geographic region. Forest plot showing the pooled proportion of any cardiac arrhythmia among patients with dengue, stratified by geographic region (Americas vs. Asia), using a random-effects generalized linear mixed model (GLMM) with logit transformation. Estimates are expressed as events per 100 patients with corresponding 95% confidence intervals. The pooled proportion was 10.07% (95% CI 4.23–22.11) in the Americas and 27.95% (95% CI 20.11–37.41) in Asia, with an overall pooled proportion of 24.48% (95% CI 17.54–33.07). The test for subgroup differences was statistically significant ($Chi^2 = 5.64$, df = 1, $p = 0.0176$). The studies included in this analysis correspond to references [12–46].

F

2. For lab confirmation

Author	Year	Event	Total	Weight	Proportion	95% CI
**Lab confirmation**						
Arfeen	2024	9	78	.	11.54	[5.41; 20.78]
Baqi	2022	25	1008	.	2.48	[1.61; 3.64]
Bhatt	2020	40	182	.	21.98	[16.19; 28.70]
Budhia	2024	15	60	.	25.00	[14.72; 37.86]
Buntubatu	2019	17	45	.	37.78	[23.77; 53.46]
Cabrera−Rego	2021	72	427	.	16.86	[13.43; 20.76]
Chaudhary	2020	5	80	.	6.25	[2.06; 13.99]
Dhivya	2019	37	250	.	14.80	[10.64; 19.82]
Furlan−Daniel	2019	7	93	.	7.53	[3.08; 14.90]
Garg	2024	100	385	.	25.97	[21.66; 30.66]
Godoy	2018	20	310	.	6.45	[3.99; 9.79]
Gugale	2022	24	140	.	17.14	[11.30; 24.42]
Gupta	2022	93	150	.	62.00	[53.72; 69.79]
Kollipara	2025	45	100	.	45.00	[35.03; 55.27]
Kothendaraman	2024	32	101	.	31.68	[22.78; 41.69]
Kularatne	2007	75	120	.	62.50	[53.20; 71.17]
Kumari	2024	45	110	.	40.91	[31.63; 50.69]
La−Fontaine−Terry	2023	28	1037	.	2.70	[1.80; 3.88]
La−Orkhun	2011	10	35	.	28.57	[14.64; 46.30]
Li	2016	113	201	.	56.22	[49.06; 63.19]
Nadkarni	2020	8	75	.	10.67	[4.72; 19.94]
Nerella	2022	62	150	.	41.33	[33.36; 49.65]
Pothapregada	2016	5	254	.	1.97	[0.64; 4.53]
Ramchandwani	2024	10	40	.	25.00	[12.69; 41.20]
Ruhella	2018	87	221	.	39.37	[32.88; 46.14]
Salam	2022	34	100	.	34.00	[24.82; 44.15]
Shah	2020	112	320	.	35.00	[29.78; 40.50]
Tabassum	2023	12	50	.	24.00	[13.06; 38.17]
Tarique	2013	26	116	.	22.41	[15.19; 31.09]
Thirumurugan	2022	34	100	.	34.00	[24.82; 44.15]
Vikas	2023	25	100	.	25.00	[16.88; 34.66]
**Total (95% CI)**		**1227**	**6438**	**.**	**21.42**	**[15.43; 28.93]**
Heterogeneity: $Tau^2 = 1.2328$; $Chi^2 = 843.83$, df = 30 ($P < 0.0001$); $I^2 = 96.4\%$						
Test for overall effect: Z = . (P = .)						
**No lab confirmation**						
Brito	2014	89	245	.	36.33	[30.30; 42.69]
Gnanamuthu	2018	60	200	.	30.00	[23.74; 36.86]
Hussain	2017	24	24	.	100.00	[85.75; 100.00]
Iqbal	2023	19	41	.	46.34	[30.66; 62.58]
**Total (95% CI)**		**192**	**510**	**.**	**63.51**	**[17.96; 93.26]**
Heterogeneity: $Tau^2 = 3.7854$; $Chi^2 = 4.63$, df = 3 ($P = 0.2008$); $I^2 = 35.2\%$						
Test for overall effect: Z = . (P = .)						
**Total (95% CI)**		**1419**	**6948**	**100.0%**	**24.48**	**[17.54; 33.07]**
Heterogeneity: $Tau^2 = 1.5426$; $Chi^2 = 861.99$, df = 34 ($P < 0.0001$); $I^2 = 96.1\%$						
Test for subgroup differences: $Chi^2 = 2.96$, df = 1 ($P = 0.0853$)						

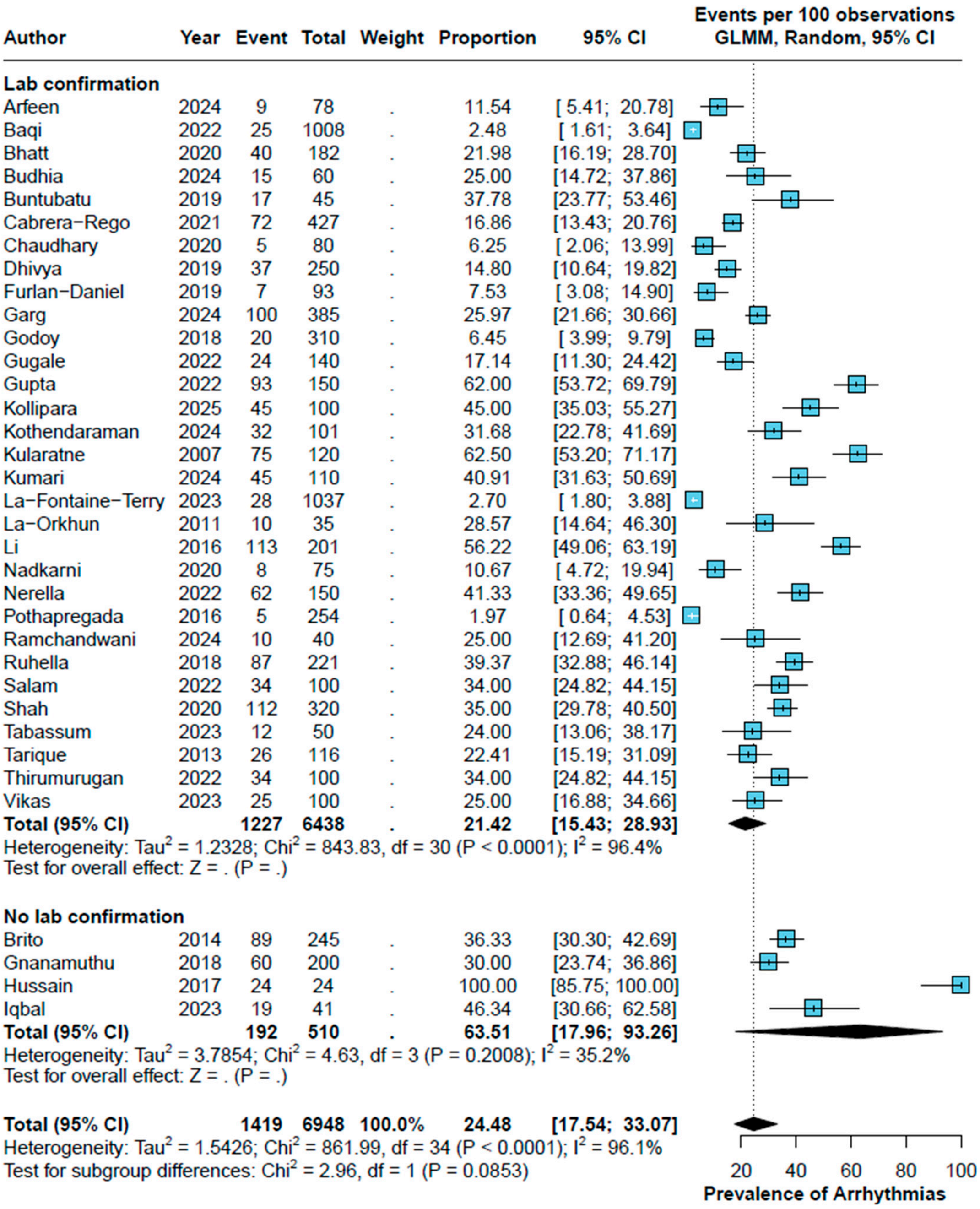

Figure S10. Subgroup analysis of the pooled proportion of any cardiac arrhythmia in dengue by laboratory confirmation status. Forest plot showing the pooled proportion of any cardiac arrhythmia among patients with dengue, stratified by laboratory confirmation status, using a random-effects generalized linear mixed model (GLMM) with logit transformation. Estimates are expressed as events per 100 patients with corresponding 95% confidence intervals. The pooled proportion was 21.42% (95% CI 15.43–28.93) in studies with laboratory confirmation and 63.51% (95% CI 17.96–93.26) in studies without clear laboratory confirmation, with an overall pooled proportion of 24.48% (95% CI 17.54–33.07). The test for subgroup differences was not statistically significant ($Chi^2 = 2.96$, df = 1, p = 0.0853). The studies included in this analysis correspond to references [12–46].

F

Analysis

Exploratory subgroup analyses were performed to assess whether geographic region and laboratory confirmation status contributed to between-study heterogeneity. Studies classified from Asia showed a higher pooled proportion of arrhythmias than those from the Americas,

although this result should be interpreted cautiously because heterogeneity remained high within both strata and at least one study included a mixed population across regions. Studies with laboratory-confirmed DENV infection showed a lower pooled proportion than those without clear laboratory confirmation, but the subgroup difference was not statistically significant. Overall, these analyses suggest that both factors may contribute to variability, but neither fully explains the substantial heterogeneity observed across studies.
